# Supplementary material for: Song flight and 3D thermal detection provide evidence for bat attraction to wind turbines in Central Europe
Source: Commun Biol. 2026 Mar 19;9:460. doi: 10.1038/s42003-026-09882-7 (PMC13032924; doi:10.1038/s42003-026-09882-7)
Supplement: Supplementary file 1 — Supplementary Information [file 42003_2026_9882_MOESM1_ESM.pdf]

Supporting Information for

Song flight and 3D thermal detection provide evidence for bat attraction to wind turbines in Central Europe

Martina Nagy[1,*], Klaus Hochradel[2], Claudia Haushalter[1], Ralph Simon[3,4], Natalie Weber[5], Oliver Behr[6] and Mirjam Knörnschild[1,7,8]

1 Museum für Naturkunde, Leibniz-Institute for Evolution and Biodiversity Science, Berlin, Germany.
2 Institute of Measurement and Sensor Technology, UMIT-Private University for Health Sciences Medical Informatics and Technology GmbH, Hall in Tirol, Austria.
3 Nuremberg Zoo, Am Tiergarten 30, 90480 Nuremberg, Germany
4 CoSys-Lab, Antwerp University, 2020 Antwerp, Belgium
5 Max Planck Institute of Animal Behavior, Radolfzell, Germany
6 OekoFor GbR, Freiburg, Germany
7 Evolutionary Ethology, Institute for Biology, Humboldt-Universität zu Berlin, Germany
8 Deutsche Fledermauswarte e.V., Am Juliusturm 64, 13599 Berlin, Germany

*Martina Nagy
Email: martina.nagy@mfn.berlin

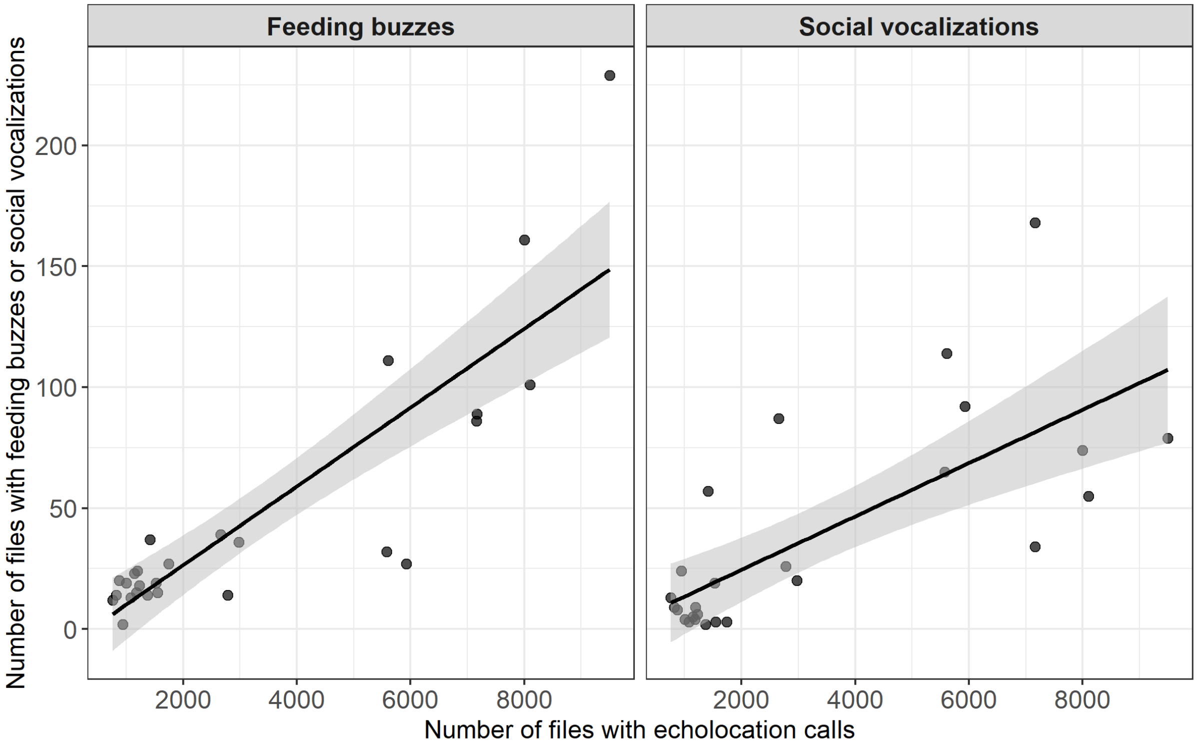

Supplementary Figure 1. Relation between feeding or social activity and overall bat activity at wind turbines.

The number of recorded files containing feeding buzzes or social vocalizations (social calls and/or song elements) was positively correlated with the number of recorded echolocation files per turbine year. Graphs depict original data points with the fitted regression lines and 95 % confidence intervals (grey shaded region) for n = 26 turbine years.

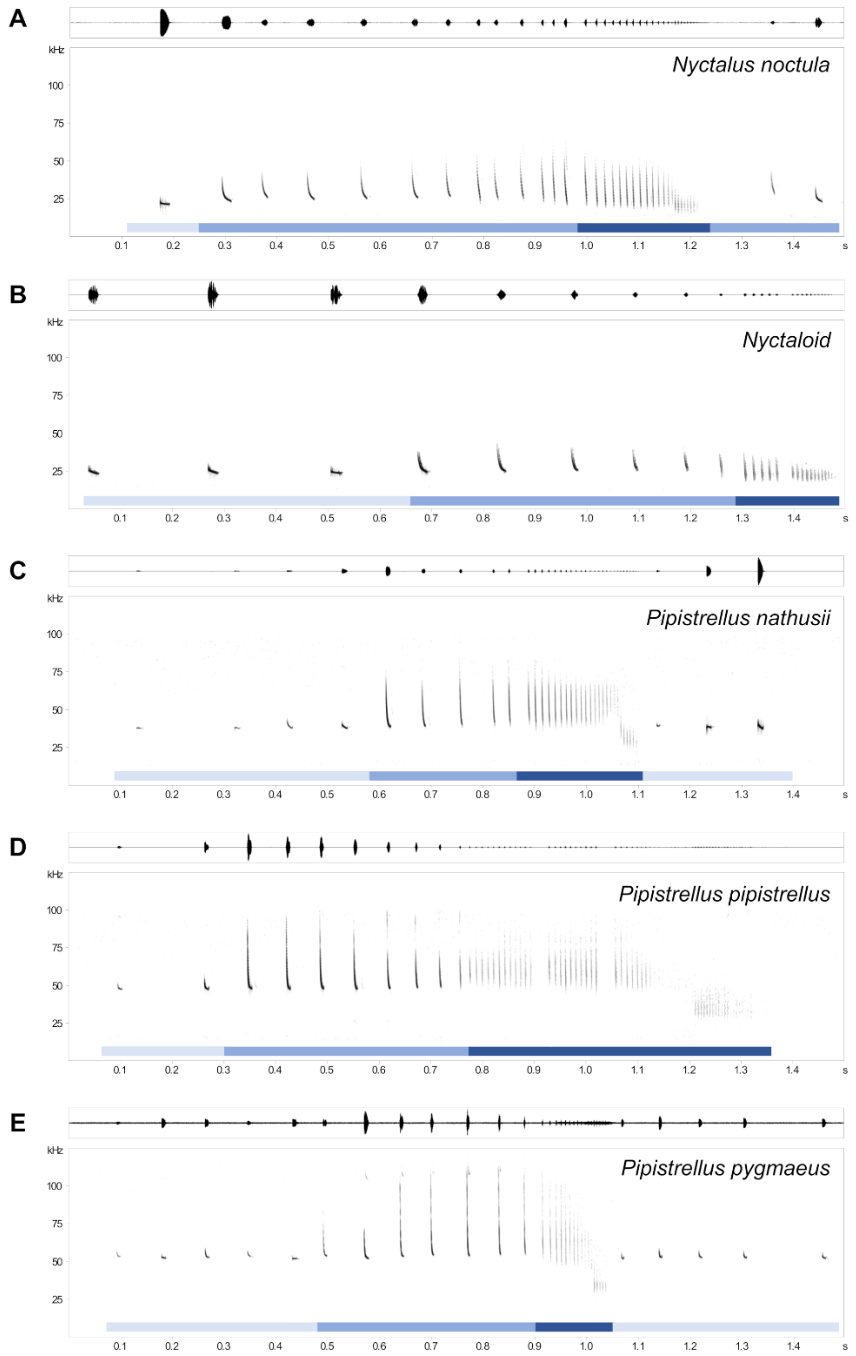

Supplementary Figure 2. Echolocation call sequences with final buzzes from five bat species (-groups) foraging in direct vicinity to wind turbines.

Sequences contain search phase calls (light blue bar), approach phase calls (middle blue bar), and final buzzes (dark blue bar). (A) *N. noctula*. (B) Nyctaloid (*N. leisleri*, *V. murinus*, *E. serotinus* or *E. nilssonii*). (C) *P. nathusii*. (D) *P. pipistrellus.* (E) *P. pygmaeus*.

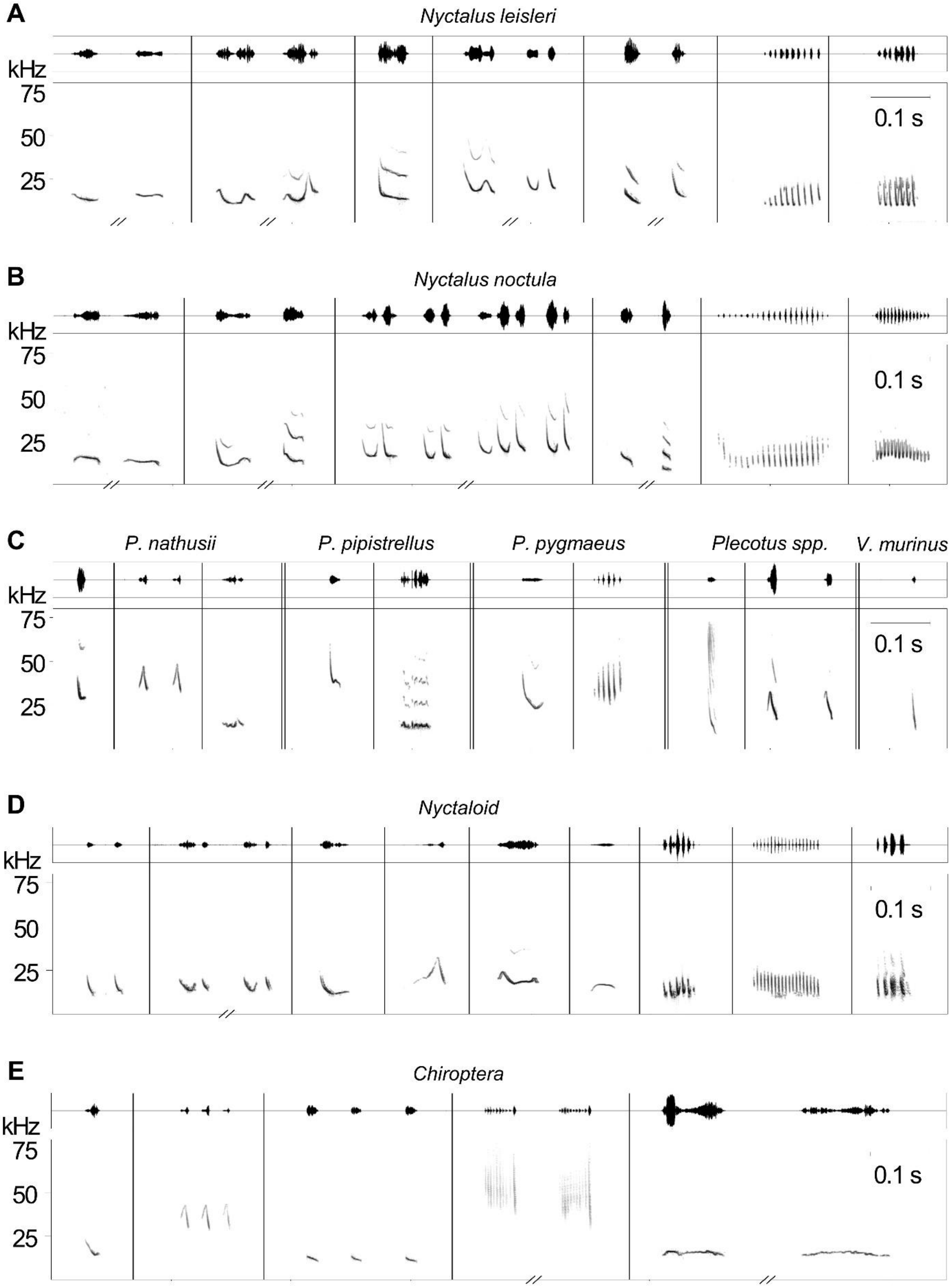
A
Nyctalus leisleri
kHz
75
50
25
0.1 s
B
Nyctalus noctula
kHz
75
50
25
0.1 s
C
P. nathusii
P. pipistrellus
P. pygmaeus
Plecotus spp.
V. murinus
kHz
75
50
25
0.1 s
D
Nyctaloid
kHz
75
50
25
0.1 s
E
Chiroptera
kHz
75
50
25
0.1 s

Supplementary Figure 3. Social vocalization types other than song from seven bat species recorded in direct vicinity to wind turbines.

Vocalizations were visually grouped into types based on their spectro-temporal appearance. Removed silent intervals are indicated as a gap in the time axis. (**A**) Seven vocalization types from *Nyctalus leisleri*: 2 long QCF, 2 QCF-inverted V, 1 L-shaped call, 2V/W motif, 2 FM down-sweep, 1 buzz, 1 long trill. (**B**) Six vocalization types from *N. noctula*: 2 long QCF, 2 L-shaped call, 2 V/W motif, 2 FM down-sweep, 1 buzz, 1 long trill. (**C**) Vocalization types from five species. *Pipistrellus nathusii*: 1 FM down-sweep, 2 inverted V, 1 warble. *P. pipistrellus*: 1 FM down-sweep, 1 warble. *P. pygmaeus*: 1 FM down-sweep, 1 buzz. *Plecotus spp.*: 1 FM down-sweep, 2 hooks. *V. murinus*: 1 FM down-sweep. Abbreviations: QCF, quasi-constant-frequency; FM, frequency modulated. (**D**) Vocalization types from Nyctaloid species: 2 FM down-sweeps, 2 V/W motif, 1 L-shaped call, 1 inverted V, 1 FM-QCF-FM, 1 QCF, 1 trill, 1 buzz, 1 noise burst. (**E**) Vocalization types from Chiroptera: 1 FM down-sweep, 3 inverted V, 3 short QCF, 2 buzz, 2 long QCF trills.

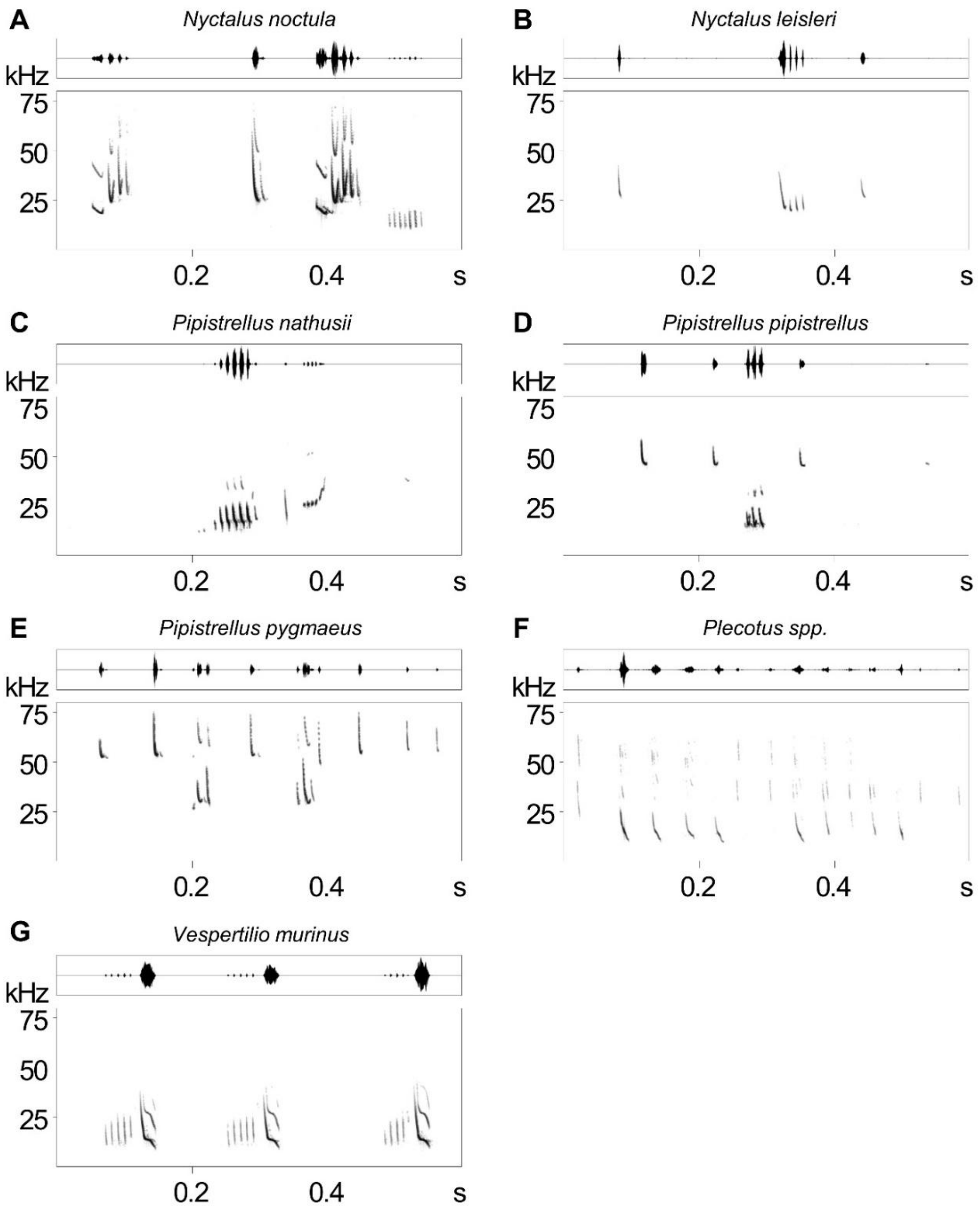

Supplementary Figure 4. Song motifs/syllables from seven bat species recorded in direct vicinity to wind turbines.

(A) Two song motifs from *N. noctula*. (B) One song motif from *N. leisleri*. (C) One song motif from *P. nathusii*. (D) One song motif from *P. pipistrellus*. (E) Two song motifs from *P. pygmaeus*. (F) Five song syllables from *Plecotus spp.* (G) Three song motifs from *V. murinus*.

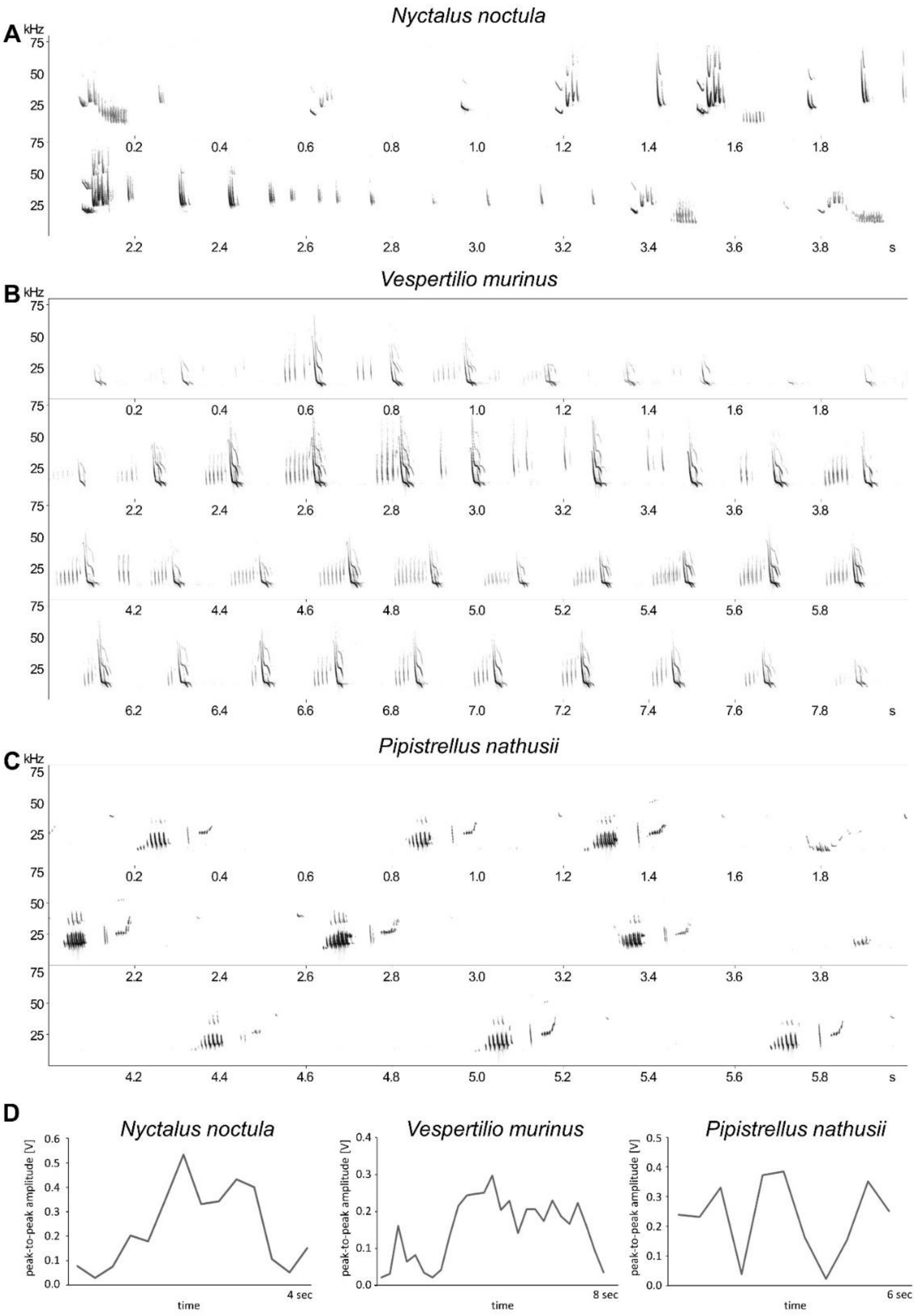

Supplementary Figure 5. Song sequences from three bat species flying in direct vicinity to wind turbines.

(A) Seven song motifs from *N. noctula*. (B) 40 song motifs from *V. murinus*. (C) Eleven song motifs from *P. nathusii*. (D) Some elements of the song are much fainter than others (i.e. have a lower peak-to-peak amplitude), presumably because the singing bat was temporarily flying away from the microphone.

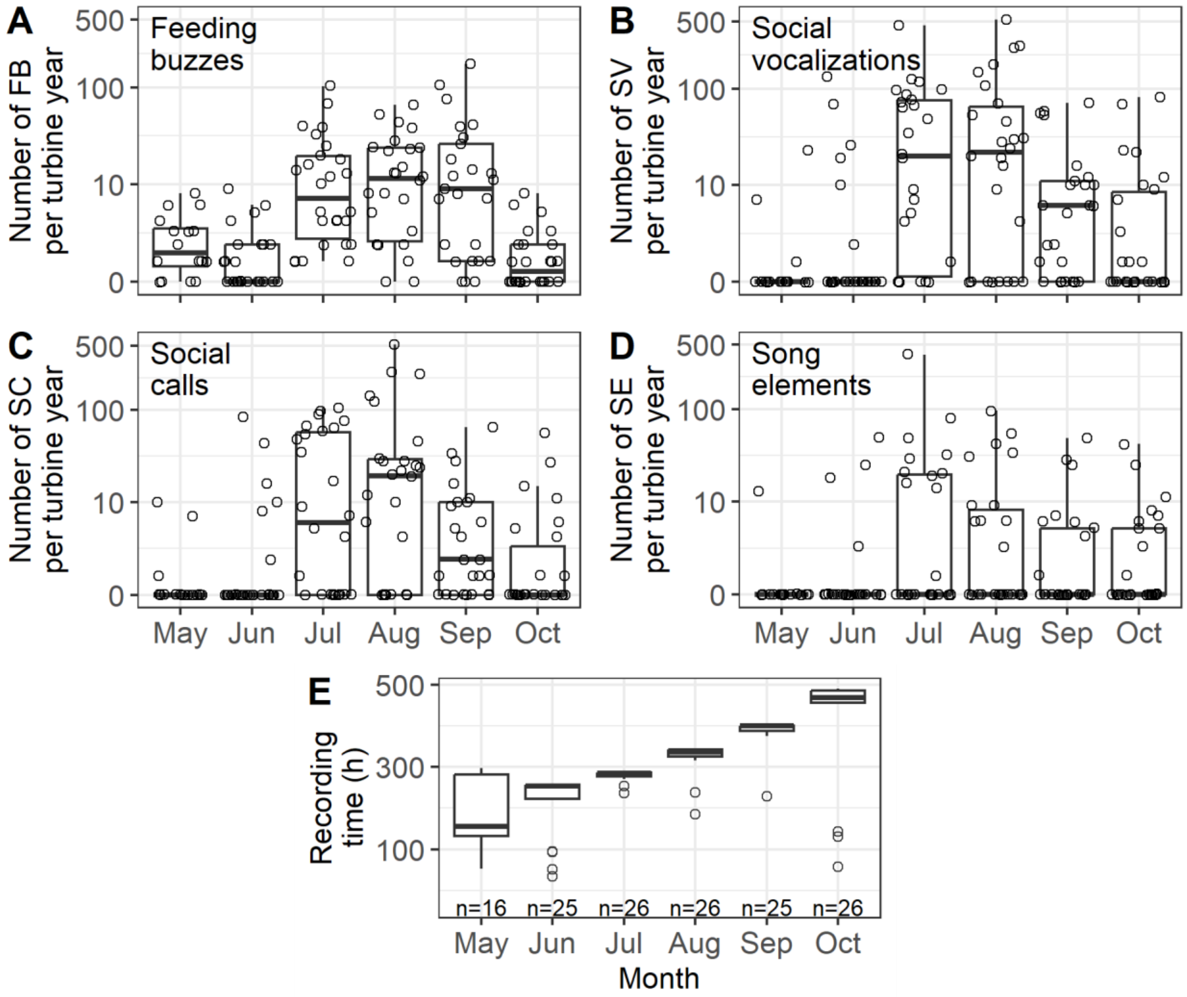

Supplementary Figure 6. Temporal distribution of feeding and social activity at wind turbines.

Boxplots (A-D) show the median number of recorded (A) feeding buzzes, (B) social vocalizations (the sum of social calls and song elements), (C) social calls, and (D) song elements per turbine year and month. Whiskers extend to 1.5x the interquartile range (IQR). Open circles depict the original data per turbine year and month. Panels A – D have a pseudo-logarithmic y-axis. (E) Boxplots show median recording hours per turbine year and month and indicated sample size corresponds to the number of turbine years. Please note that because nights are shortest in June, the increase in recording time from June to October reflects the fact that nights are getting longer as the year progresses. However, the lower recording time for May represents a lower number of sampling nights for this month. FB: feeding buzzes, SV: social vocalizations, SC: social calls, SE: song elements.

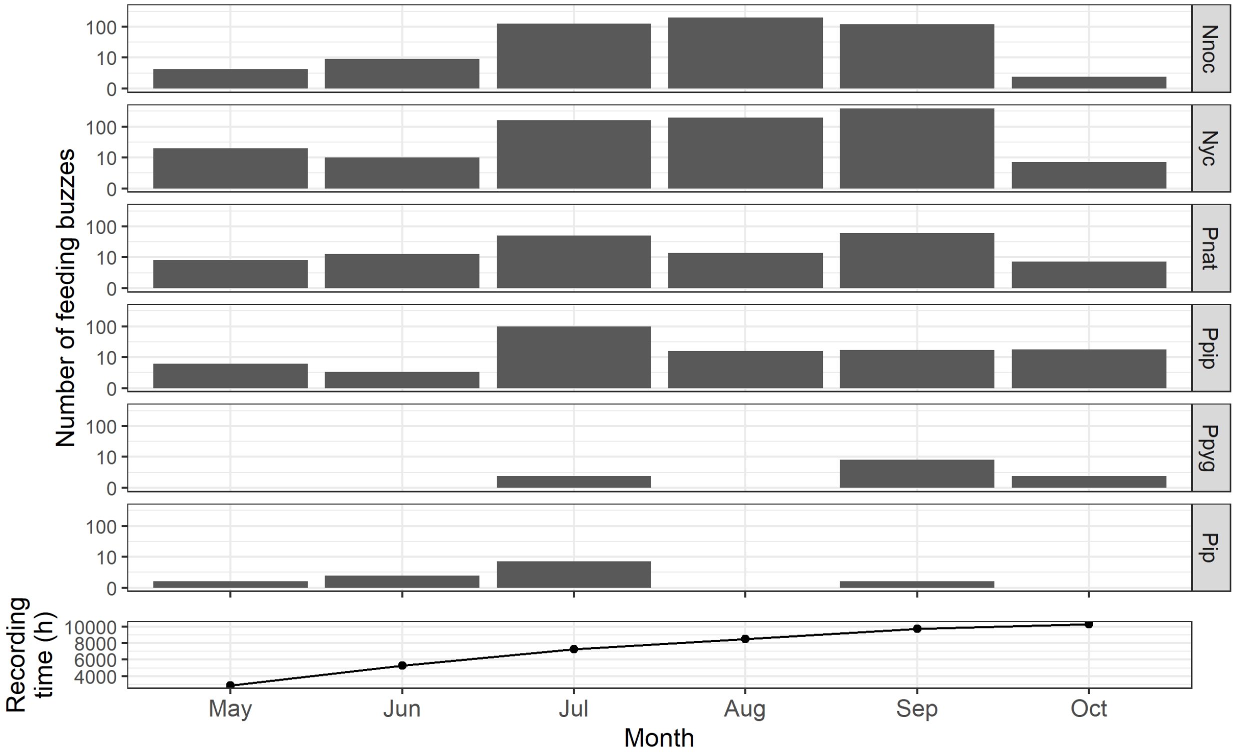

Supplementary Figure 7. Temporal distribution of feeding activity across species (-groups).

The upper panel shows the total number of feeding buzzes detected per species (-group) and month. The upper panel has a pseudo-logarithmic y-axis. The lower panel shows the total recording time per month. Please note that because nights are shortest in June, the increase in recording time from June to October reflects the fact that nights are getting longer as the year progresses. However, the lower recording time for May represents a lower number of sampling nights for this month. Nnoc: *N. noctula*, Nyc: Nyctaloid bat species, Pnat: *P. nathusii*, Ppip: *P. pipistrellus*, Ppyg: *P. pygmaeus*, Pip: Pipistrelloid bat species.

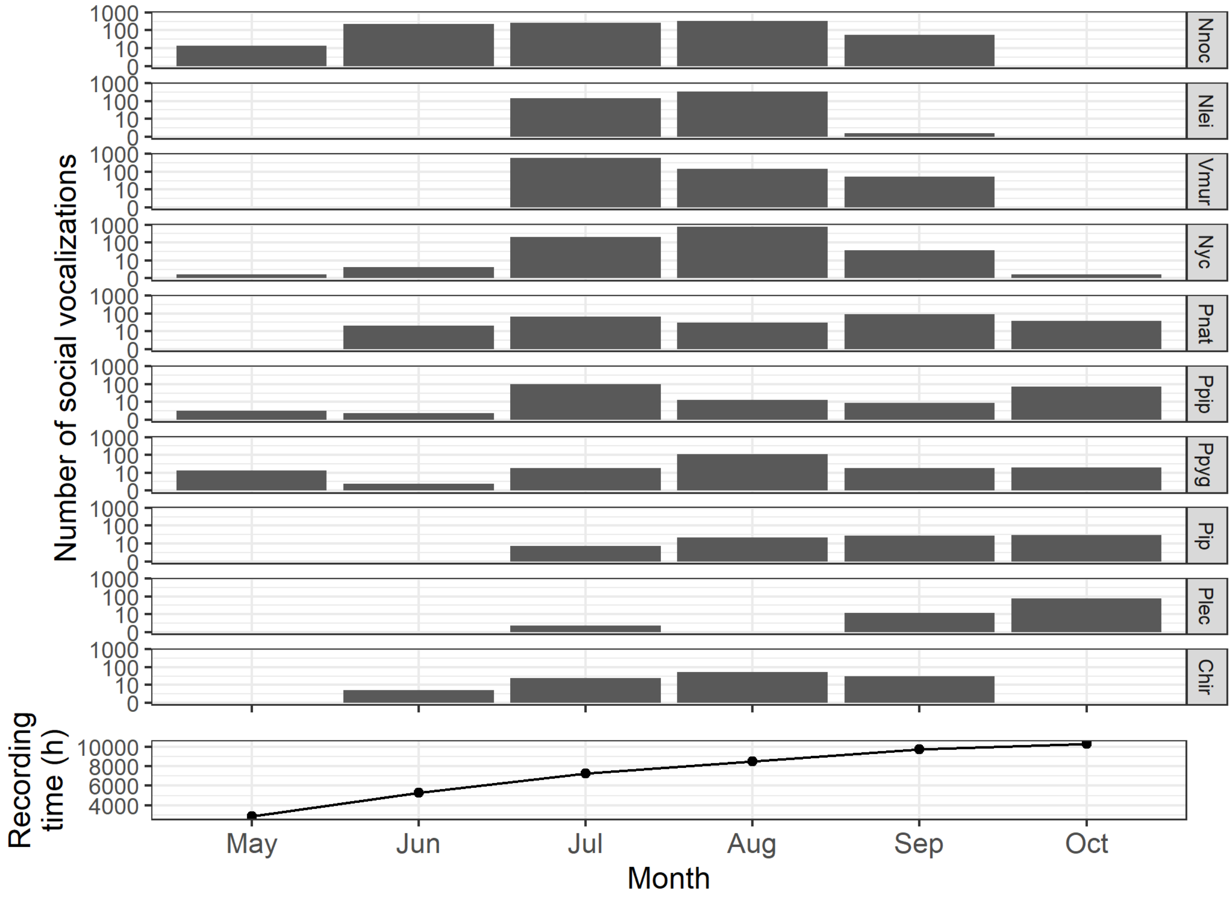

Supplementary Figure 8. Temporal distribution of social activity across species (-groups).

The upper panel shows the total number of social vocalizations (social calls and songs) detected per species (-group) and month. The upper panel has a pseudo-logarithmic y-axis. The lower panel shows the total recording time per month. Please note that because nights are shortest in June, the increase in recording time from June to October reflects the fact that nights are getting longer as the year progresses. However, the lower recording time for May represents a lower number of sampling nights for this month. Nnoc: *N. noctula*, Nlei: *N. leisleri*, Vmur: *V. murinus*, Nyc: Nyctaloid bat species, Pnat: *P. nathusii*, Ppip: *P. pipistrellus*, Ppyg: *P. pygmaeus*, Pip: Pipistrelloid bat species, Plec: *Plecotus spp.*, Chir: Chiroptera (unidentified bat).

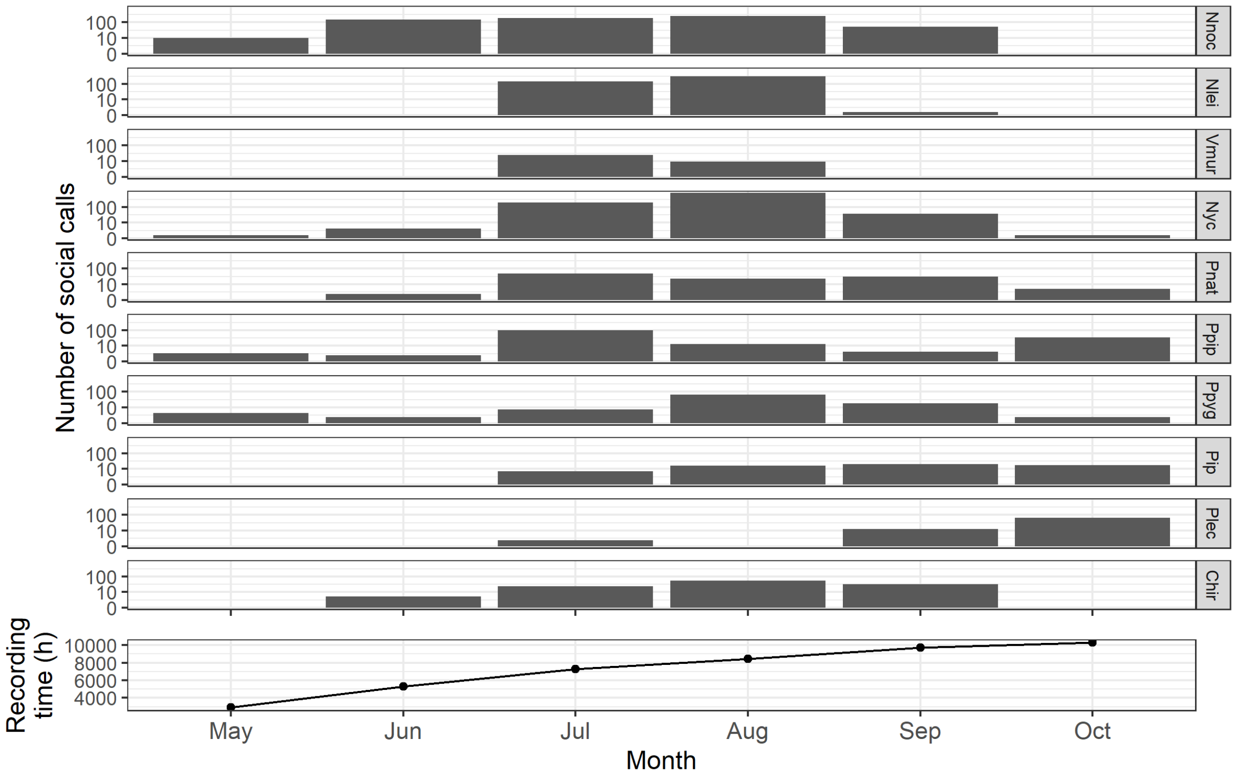

Supplementary Figure 9. Temporal distribution of social calls across species (-groups).

The upper panel shows the total number of social calls detected per species (-group) and month. The upper panel has a pseudo-logarithmic y-axis. The lower panel shows the total recording time per month. Please note that because nights are shortest in June, the increase in recording time from June to October reflects the fact that nights are getting longer as the year progresses. However, the lower recording time for May represents a lower number of sampling nights for this month. Nnoc: *N. noctula*, Nlei: *N. leisleri*, Vmur: *V. murinus*, Nyc: Nyctaloid bat species, Pnat: *P. nathusii*, Ppip: *P. pipistrellus*, Ppyg: *P. pygmaeus*, Pip: Pipistrelloid bat species, Plec: *Plecotus spp.*, Chir: Chiroptera (unidentified bat).

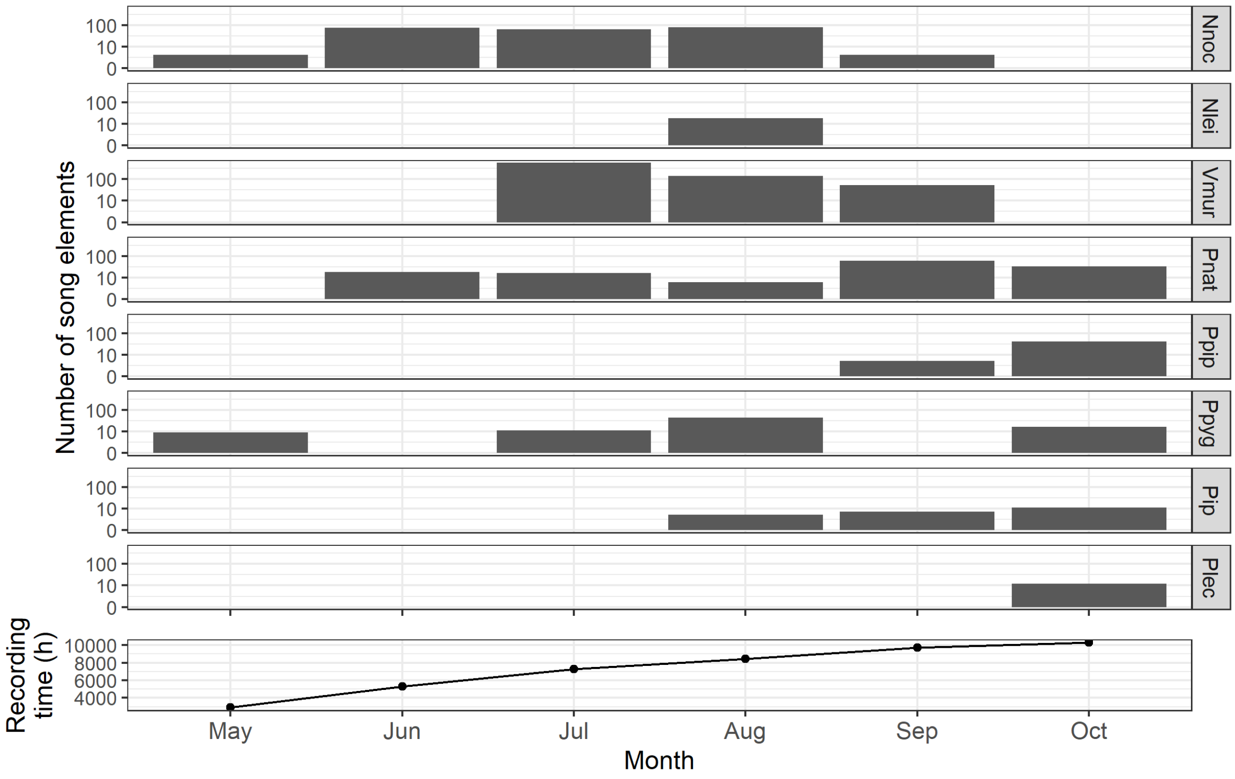

Supplementary Figure 10. Temporal distribution of bat songs across species (-groups).

The upper panel shows the total number of song elements per species (-group) and month. The lower panel shows the total recording time per month. Please note that because nights are shortest in June, the increase in recording time from June to October reflects the fact that nights are getting longer as the year progresses. However, the lower recording time for May represents a lower number of sampling nights for this month. Nnoc: *N. noctula*, Nlei: *N. leisleri*, Vmur: *V. murinus*, Pnat: *P. nathusii*, Ppip: *P. pipistrellus*, Ppyg: *P. pygmaeus*, Pip: Pipistrelloid bat species, Plec: *Plecotus spp.*.

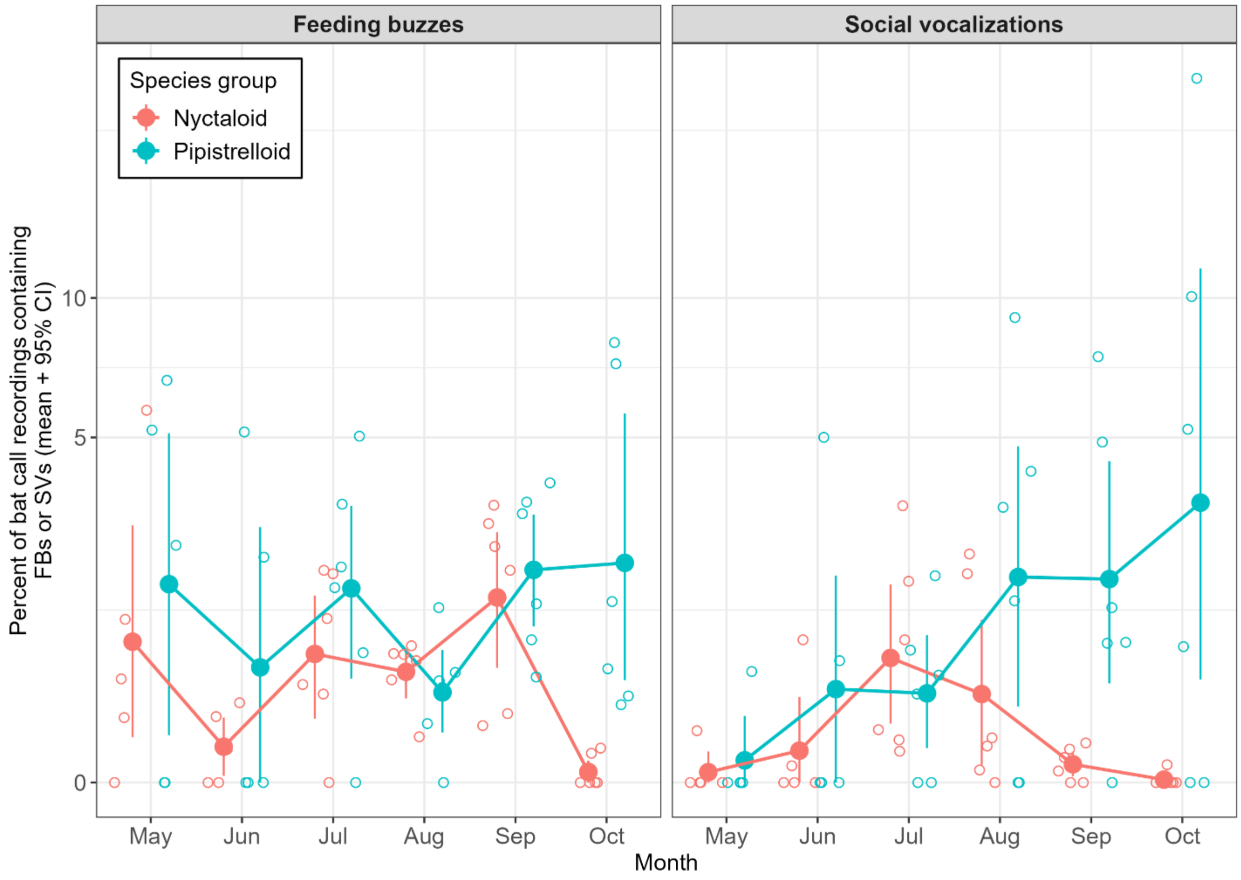

Supplementary Figure 11. Monthly proportions of bat call recordings containing feeding buzzes or social vocalizations.

Graphs show the monthly mean and 95% bootstrap confidence interval proportion of Nyctaloid or Pipistrelloid call recordings that contained feeding buzzes or social vocalizations (social calls and/or songs) of the respective species group. Open circles show the underlying data and are means per month and site to account for non-independence of data from turbines of the same site. Due to a low overall bat activity in October, proportions of Pipistrelloid feeding and social activity vary widely between sites and means may be subject to much larger uncertainty. Panels have a pseudo-logarithmic y-axis. n = 6 biologically independent wind turbine sites.

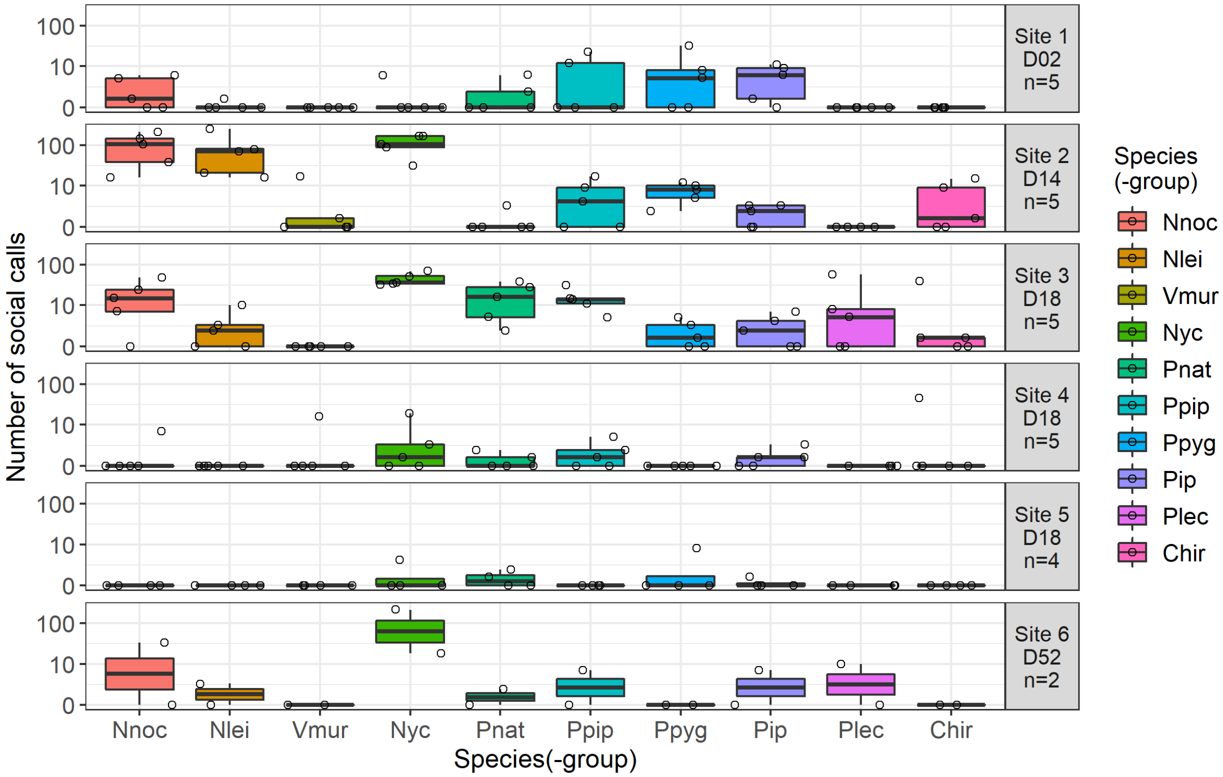

Supplementary Figure 12. Occurrence of social calls at wind turbines.

Boxplots show the median number of recorded social calls per species (-group) and site. Whiskers extend to 1.5x the interquartile range (IQR). Open circles depict the original data per species (-group) and turbine year. Panel labels report site numbers, the natural regions of Germany where wind turbine sites were located and the number of sampled turbine years per site (n = 5, 5, 5, 5, 4, and 2; total n = 26 wind turbine-years from 6 biologically independent wind turbine sites). Panels have a pseudo-logarithmic y-axis. Species (-group) abbreviations: Nnoc, *N. noctula*; Nlei, *N. leisleri*; Vmur, *V. murinus*; Nyc, Nyctaloid species; Pnat, *P. nathusii*; Ppip, *P. pipistrellus*; Ppyg, *P. pygmaeus*; Pip, *Pipistrellus spp.*; Plec, *Plecotus spp.*

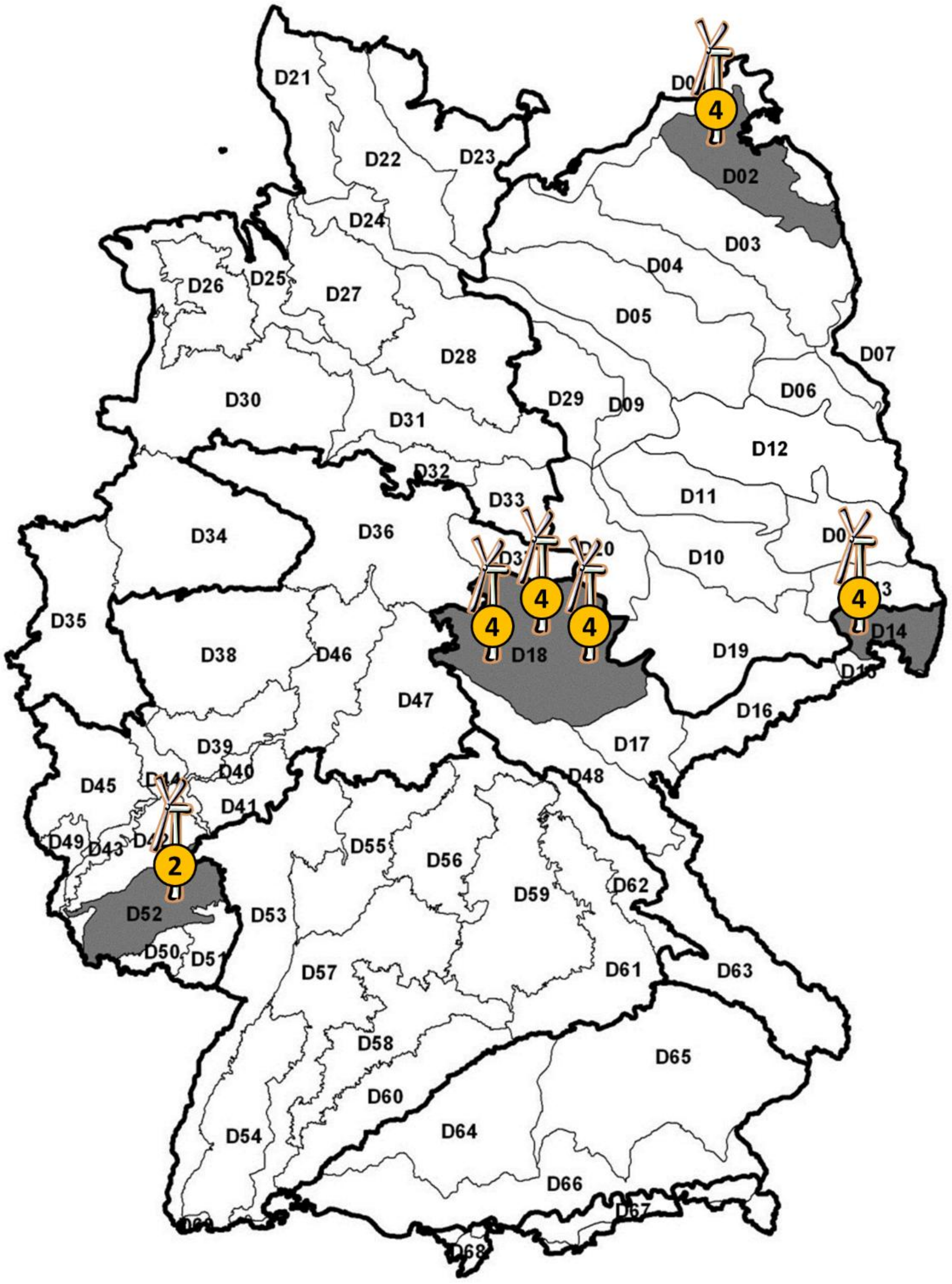

Supplementary Figure 13. Locations of wind turbines sampled in different natural regions of Germany.

Each wind turbine site is represented by a wind turbine symbol and numbers in orange circles indicate the number of sampled wind turbines per site. Wind turbine sites were sampled in four different major landscape units: D02 Northeast Mecklenburg Plain (site 1), D14 Upper Lusatia (site 2), D18 Thuringian Basin (site 3-5), D52 Saar-Nahe Hills (site 6). Thicker lines in the map indicate borders of the eight so-called great landscapes of Germany where D02 belongs to the Northeast German Plain, D14 and D18 belong to the Eastern Central Uplands and, D52 belongs to the Western Central Uplands.

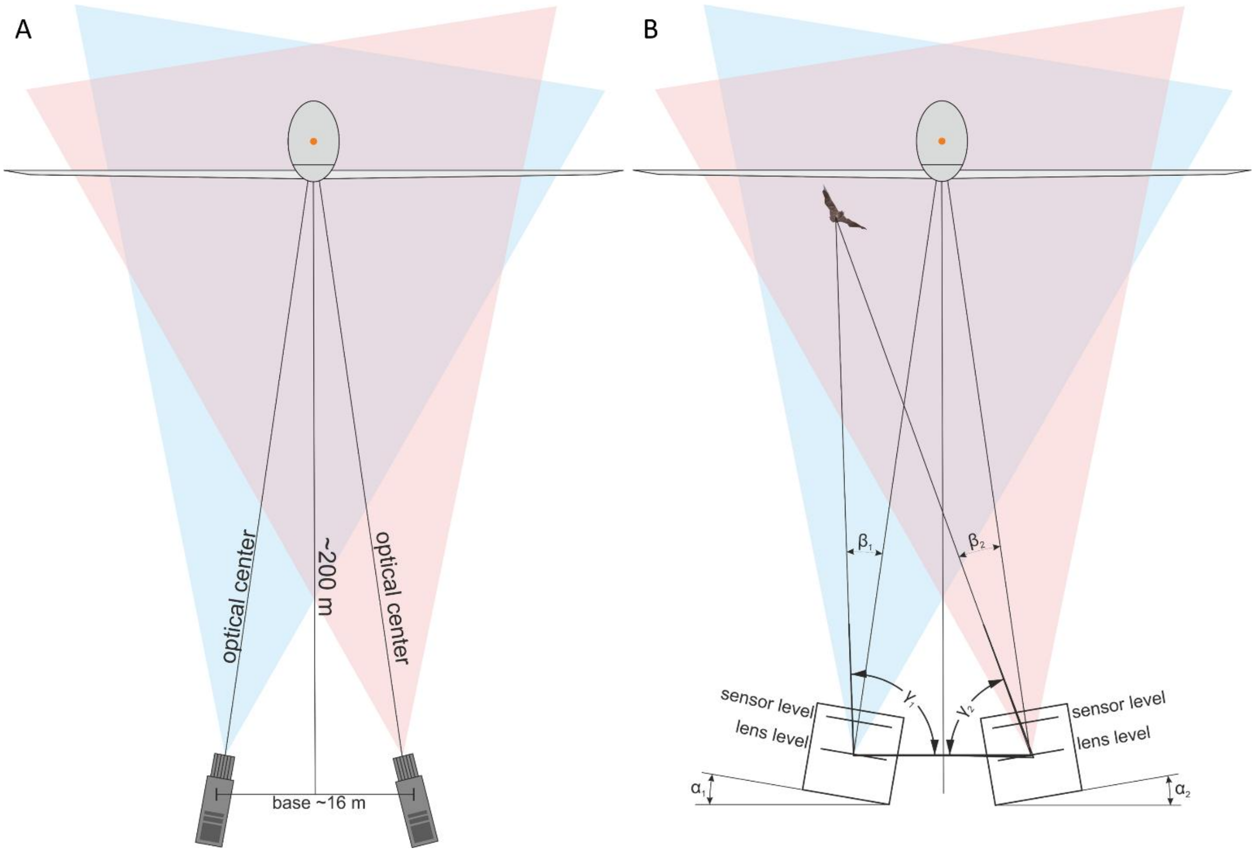

Supplementary Figure 14. Experimental setup of the two thermal cameras relative to the wind turbine.

(A) Two cameras were positioned 200 m from the wind turbine, with a baseline distance of approximately 16 m. Both cameras were aligned to capture the same reference point on top of the turbine's nacelle (orange dot), maximizing the overlapping field of view at the turbine's position. (B) The position of an unknown object (e.g., a bat) in space is determined using trigonometric calculations. Known distances, measured with a laser rangefinder, provide the angles α1 and α2 which are essential for solving the trigonometric equations. The angles β are derived from image analysis, considering the known focal length (i.e., the distance between the sensor and lens).

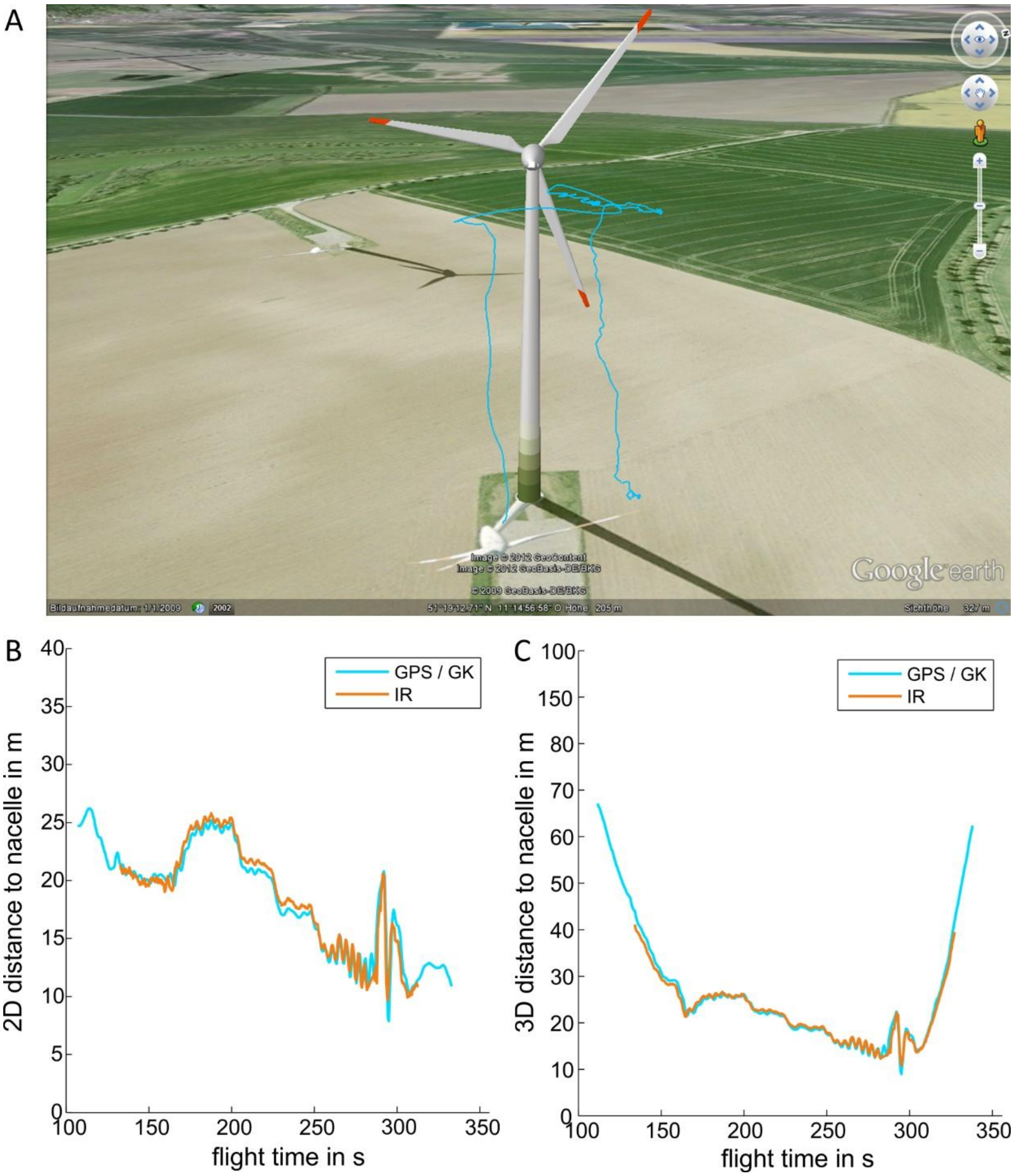

Supplementary Figure 15. Experimental drone flights to investigate accuracy and systematic errors.

Multiple drone flights were conducted parallel to the deployment of the stereo thermal camera system. (A) Example of a three-dimensional flight path of a GPS-equipped drone. Drone flights were conducted to evaluate the localization accuracy of the stereo thermal camera system. (B) Comparison of the two-dimensional distance between the drone and the nacelle, based on GPS data (blue line) and stereo thermal triangulation results (golden line). (C) Comparison of the three-dimensional distance between the drone and the nacelle, using GPS data (blue line) and stereo thermal triangulation results (golden line). No systematic errors were detected. Mismatches illustrated by non-overlapping sections of the blue and gold lines resulted from the accuracy of the GPS and two-dimensional detection error in determining the drone's center.

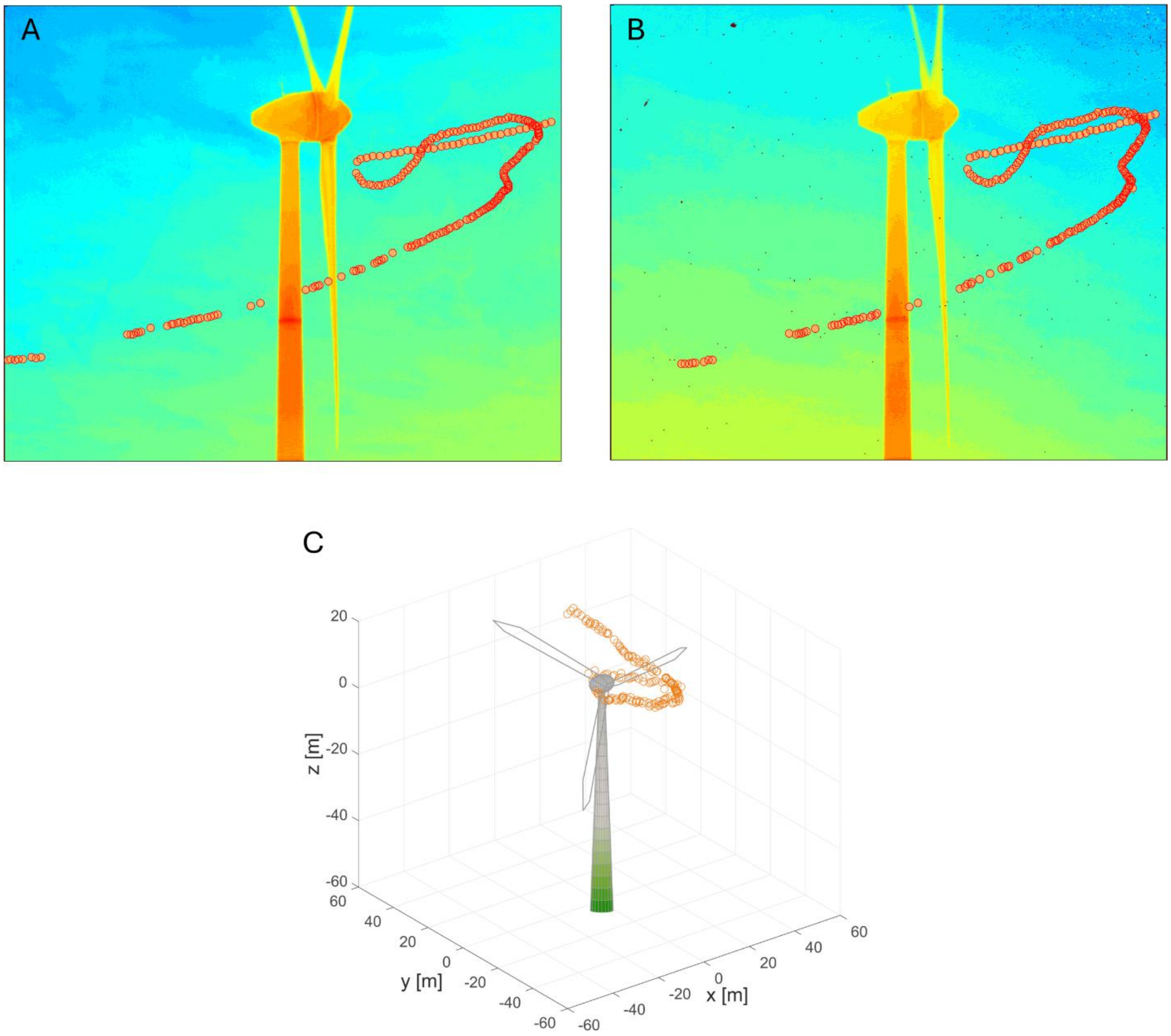

Supplementary Figure 16. Stereo thermal recordings of a suspected inspection flight trajectory of a bat within the rotor swept zone of a wind turbine

The images display the reconstructed 2D flight paths captured by two thermal cameras (A and B), along with the 3D flight trajectory derived from triangulation (C). The rotor position is exemplary.

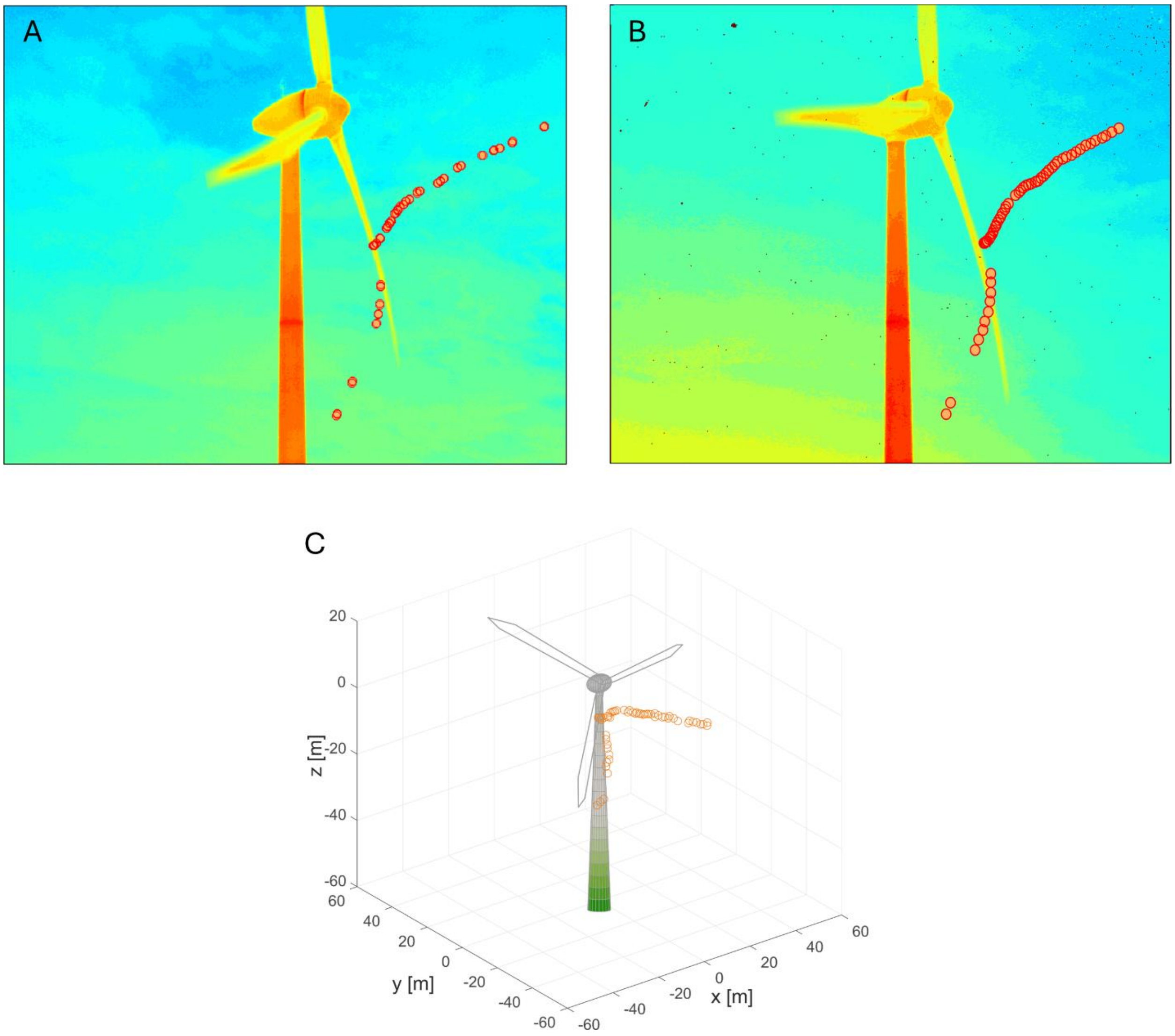

Supplementary Figure 17. Stereo thermal recordings of a suspected bat collision by the rotor blade of a wind turbine

The images display the reconstructed 2D flight paths captured by two thermal cameras (A and B), along with the 3D flight trajectory derived from triangulation (C). The rotor position during the suspected collision is shown.

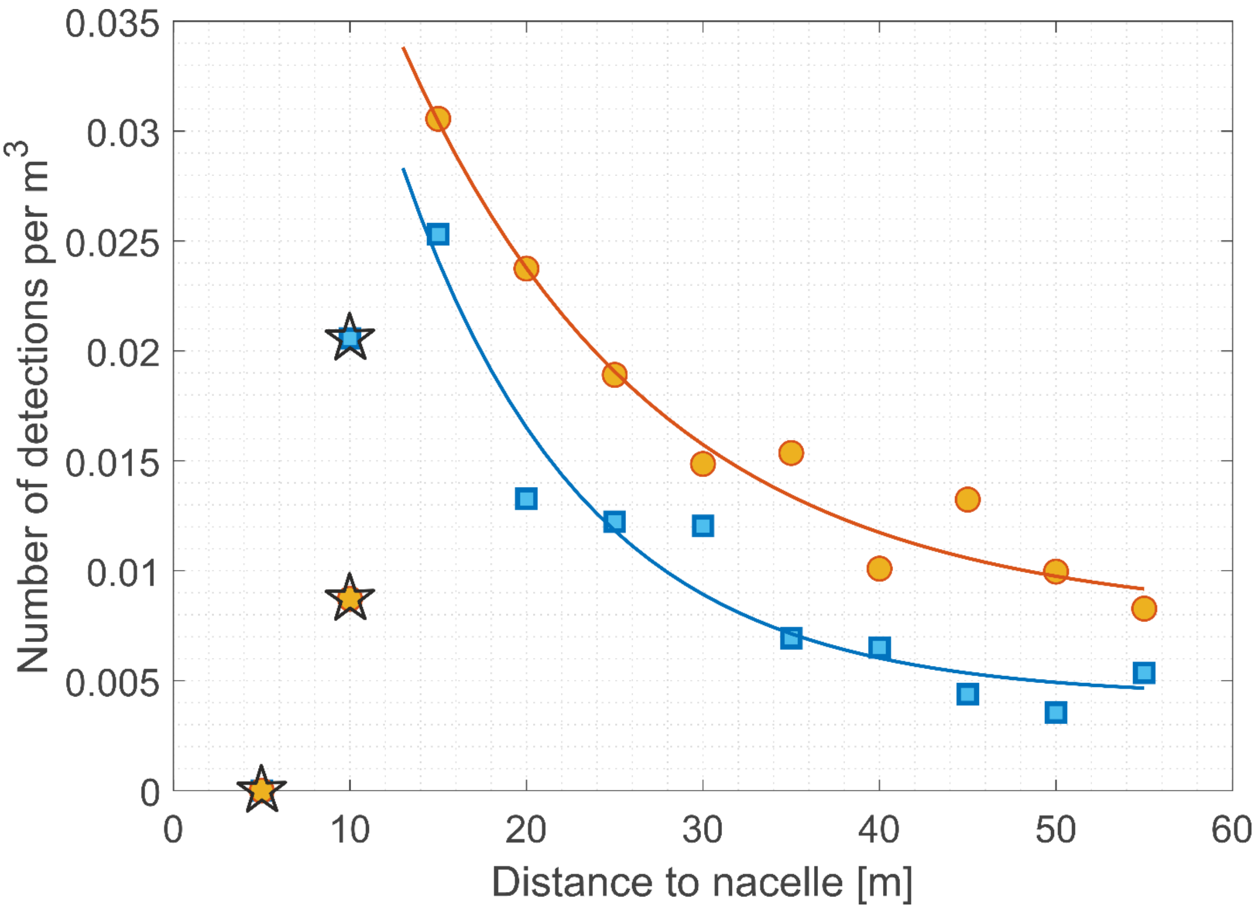

Supplementary Figure 18. Comparison of bat density in front of and behind the wind turbine.

The orange dots and curve represent bat density in front of the wind turbine, while the blue dots and curve represent bat density behind it. Although the curves are qualitatively similar, the activity levels differ, likely due to a general reduction in detection probability with increasing distance from the cameras. The nacelle occupies a large part of the volume in close vicinity to the nacelle (< 10 m, marked by orange and blue stars) explaining the low activity in this area.

Supplementary Table 1. Number of social vocalizations, social calls, song elements, and song events per species (-group)

Species	Total number of social vocalizations	Total number of social calls	Total number of song elements	Total number of song events
V. murinus	807	34	773	14
N. noctula	891	660	231	38
N. leisleri	473	455	18	1
Nyctaloid	1045	1045	0	0
P. nathusii	242	108	134	22
P. pipistrellus	201	156	45	6
P. pygmaeus	181	99	82	5
Pipistrellus spp.	84	61	23	4
Plecotus spp.	92	80	12	2
Chiroptera	113	113	0	0
**Sum**	**4129**	**2811**	**1318**	**92**

Supplementary Table 2. Number of vocalization types per species (-group)

Species	Syllable type or song element type	No. of syllables or song elements
Vespertilio murinus	FM down-sweep	34
	SONG (buzz and FM-QCF(-FM) motif)	773
Nyctalus noctula	FM down-sweep	403
	V/W motif	55
	L-shaped call	28
	long QCF	16
	buzz	78
	long trill	80
	SONG (chirps)	231
Nyctalus leisleri	FM down-sweep	177
	V/W motif	215
	L-shaped call	6
	QCF-inverted V	47
	long QCF	5
	buzz	2
	long trill	3
	SONG (short trill)	18
Nyctaloid	FM down-sweep	685
	V/W motif	95
	L-shaped call	65
	buzz	173
	noise bursts	10
	QCF	7
	FM-QCF-FM	4
	trill	4
	inverted V	2

Species	Syllable type or song element type	No. of syllables or song elements
Pipistrellus nathusii	low freq FM down-sweep	25
	inverted V	27
	trill (only motif A)	55
	warble	1
	SONG (complex trill with motifs A-B-C)	134
Pipistrellus pipistrellus	low freq FM down-sweep	96
	single trill (not in series)	59
	warble	1
	SONG (trills in series)	45
Pipistrellus pygmaeus	low freq FM down-sweep	79
	buzz	10
	single trill (not in series)	10
	SONG (trills in series)	82
Pipistrellus spp.	single trill (not in series)	84
Plecotus spp. (cf. auritus)	low freq FM down-sweep (not in series)	72
	low freq hooks	8
	SONG (low freq FM down-sweep in series)	12
Chiroptera	FM down-sweep	50
	inverted V	42
	buzz	11
	long QCF trill	6
	short QCF	4

Supplementary Table 3. Result of post hoc pairwise comparisons of estimated marginal means (EMMs) for feeding activity based on the interaction between species group (Nyctaloid (Nyc) and Pipistrelloid (Pip) and month. Results are presented as log-odds differences, along with their standard errors (SE), degrees of freedom (df), z-ratios, and p-values. Significant differences ($p < 0.05$) are highlighted in bold.

Contrast	Estimate	SE	df	z.ratio	P-value
**Contrasts between Nyc and Pip within the same months**					
Nyc Mai - Pip Mai	-0.8078	0.513	Inf	-1.574	0.9187
Nyc Jun - Pip Jun	-1.6528	0.514	Inf	-3.218	0.0583
Nyc Jul - Pip Jul	-0.7403	0.265	Inf	-2.798	0.1806
Nyc Aug - Pip Aug	-0.0715	0.314	Inf	-0.228	1
Nyc Sep - Pip Sep	-0.2865	0.272	Inf	-1.053	0.9964
Nyc Oct - Pip Oct	-2.0233	0.541	Inf	-3.743	**0.0099**
**Contrasts for Nyc species between months**					
Nyc Mai - Nyc Jun	0.999	0.476	Inf	2.098	0.6243
Nyc Mai - Nyc Jul	-0.0827	0.371	Inf	-0.223	1
Nyc Mai - Nyc Aug	0.1819	0.367	Inf	0.495	1
Nyc Mai - Nyc Sep	-0.3063	0.374	Inf	-0.82	0.9996
Nyc Mai - Nyc Oct	1.5851	0.556	Inf	2.85	0.1595
Nyc Jun - Nyc Jul	-1.0817	0.383	Inf	-2.823	0.17
Nyc Jun - Nyc Aug	-0.8171	0.38	Inf	-2.15	0.5864
Nyc Jun - Nyc Sep	-1.3053	0.386	Inf	-3.385	**0.0345**
Nyc Jun - Nyc Oct	0.5861	0.565	Inf	1.038	0.9968
Nyc Jul - Nyc Aug	0.2646	0.232	Inf	1.141	0.9928
Nyc Jul - Nyc Sep	-0.2236	0.241	Inf	-0.927	0.9989
Nyc Jul - Nyc Oct	1.6678	0.48	Inf	3.474	**0.0257**
Nyc Aug - Nyc Sep	-0.4882	0.236	Inf	-2.068	0.6458
Nyc Aug - Nyc Oct	1.4032	0.477	Inf	2.939	0.1273
Nyc Sep - Nyc Oct	1.8914	0.481	Inf	3.93	**0.0048**
**Contrasts for Pip species between months**					
Pip Mai - Pip Jun	0.154	0.556	Inf	0.277	1
Pip Mai - Pip Jul	-0.0153	0.448	Inf	-0.034	1

Pip Mai - Pip Aug	0.9182	0.481	Inf	1.911	0.7529
Pip Mai - Pip Sep	0.215	0.452	Inf	0.476	1
Pip Mai - Pip Oct	0.3696	0.497	Inf	0.743	0.9999
Pip Jun - Pip Jul	-0.1693	0.432	Inf	-0.392	1
Pip Jun - Pip Aug	0.7642	0.466	Inf	1.638	0.895
Pip Jun - Pip Sep	0.061	0.435	Inf	0.14	1
Pip Jun - Pip Oct	0.2155	0.486	Inf	0.444	1
Pip Jul - Pip Aug	0.9334	0.336	Inf	2.777	0.1898
Pip Jul - Pip Sep	0.2303	0.292	Inf	0.788	0.9998
Pip Jul - Pip Oct	0.3848	0.361	Inf	1.065	0.996
Pip Aug - Pip Sep	-0.7032	0.341	Inf	-2.06	0.6512
Pip Aug - Pip Oct	-0.5486	0.402	Inf	-1.364	0.9701
Pip Sep - Pip Oct	0.1545	0.366	Inf	0.423	1

Supplementary Table 4. Result of post hoc pairwise comparisons of estimated marginal means (EMMs) for social activity based on the interaction between species group (Nyctaloid (Nyc) and Pipistrelloid (Pip) and month. Results are presented as log-odds differences, along with their standard errors (SE), degrees of freedom (df), z-ratios, and p-values. Significant differences ($p < 0.05$) are highlighted in bold.

Contrast	Estimate	SE	df	z.ratio	P-value
**Contrasts between Nyc and Pip within the same months**					
Nyc Mai - Pip Mai	-1.4857	1.16	Inf	-1.284	0.9812
Nyc Jun - Pip Jun	-0.5705	0.909	Inf	-0.628	1
Nyc Jul - Pip Jul	-0.2157	0.497	Inf	-0.434	1
Nyc Aug - Pip Aug	-1.3872	0.481	Inf	-2.884	0.1464
Nyc Sep - Pip Sep	-2.5968	0.543	Inf	-4.786	**0.0001**
Nyc Oct - Pip Oct	-5.0533	1.19	Inf	-4.245	**0.0013**
**Contrasts for Nyc species between months**					
Nyc Mai - Nyc Jun	-1.2778	0.991	Inf	-1.289	0.9807
Nyc Mai - Nyc Jul	-2.1862	0.908	Inf	-2.407	0.4009
Nyc Mai - Nyc Aug	-1.6557	0.908	Inf	-1.824	0.8053
Nyc Mai - Nyc Sep	-0.4212	0.947	Inf	-0.445	1
Nyc Mai - Nyc Oct	1.3238	1.39	Inf	0.95	0.9986
Nyc Jun - Nyc Jul	-0.9084	0.627	Inf	-1.448	0.9539
Nyc Jun - Nyc Aug	-0.3778	0.628	Inf	-0.602	1
Nyc Jun - Nyc Sep	0.8566	0.681	Inf	1.257	0.9841
Nyc Jun - Nyc Oct	2.6016	1.23	Inf	2.11	0.6158
Nyc Jul - Nyc Aug	0.5306	0.452	Inf	1.174	0.9909
Nyc Jul - Nyc Sep	1.765	0.52	Inf	3.395	**0.0334**
Nyc Jul - Nyc Oct	3.51	1.17	Inf	3.008	0.106
Nyc Aug - Nyc Sep	1.2345	0.523	Inf	2.359	0.4343
Nyc Aug - Nyc Oct	2.9794	1.17	Inf	2.554	0.3062
Nyc Sep - Nyc Oct	1.745	1.2	Inf	1.458	0.9516
**Contrasts for Pip species between months**					
Pip Mai - Pip Jun	-0.3627	1.1	Inf	-0.331	1
Pip Mai - Pip Jul	-0.9162	0.9	Inf	-1.018	0.9973

Pip Mai - Pip Aug	-1.5572	0.89	Inf	-1.75	0.8448
Pip Mai - Pip Sep	-1.5324	0.902	Inf	-1.699	0.8693
Pip Mai - Pip Oct	-2.2439	0.911	Inf	-2.462	0.3642
Pip Jun - Pip Jul	-0.5535	0.83	Inf	-0.667	1
Pip Jun - Pip Aug	-1.1945	0.821	Inf	-1.454	0.9525
Pip Jun - Pip Sep	-1.1697	0.824	Inf	-1.42	0.96
Pip Jun - Pip Oct	-1.8812	0.836	Inf	-2.251	0.5119
Pip Jul - Pip Aug	-0.641	0.523	Inf	-1.226	0.987
Pip Jul - Pip Sep	-0.6162	0.527	Inf	-1.17	0.9912
Pip Jul - Pip Oct	-1.3277	0.548	Inf	-2.422	0.3907
Pip Aug - Pip Sep	0.0248	0.509	Inf	0.049	1
Pip Aug - Pip Oct	-0.6867	0.531	Inf	-1.293	0.9802
Pip Sep - Pip Oct	-0.7115	0.528	Inf	-1.347	0.9729

Supplementary Table 5. Characteristics of bat species recorded at wind turbines in Germany

Species	Typical foraging niche * †	Mating sites	Main peak of singing and mating activity	Male song production	Typical migration behavior (in Germany) †	References (for vocalizations and mating behaviour) ‡
V. murinus	Open-space forager, fast aerial hawking in uncluttered airspace	in mating roost (cliff, tall building)	October - December	in flight	predominantly migratory	[1, 2]
N. noctula	Open-space forager, fast aerial hawking in uncluttered airspace	in mating roost (tree hole, bat box)	August - September	stationary, in flight	migratory	[3, 4]
N. leisleri	Open-space forager, fast aerial hawking in uncluttered airspace	in mating roost (tree hole, bat box)	August - September	stationary, in flight	migratory	[5, 6]
P. nathusii	Edge-space forager, aerial hawking in background-cluttered airspace	in mating roost (tree hole, bat box)	August - September	stationary, in flight	migratory	[7, 8]
P. pipistrellus	Edge-space forager, aerial hawking in background-cluttered airspace	in mating roost (tree hole, bat box)	August - September	Predominantly in flight	predominantly sedentary	[9, 10]
P. pygmaeus	Edge-space forager, aerial hawking in background-cluttered airspace	in mating roost (tree hole, bat box)	August - September	Predominantly in flight	migratory, sedentary	[10, 11]
Plecotus spp.	Narrow-space forager, gleaning and aerial hawking in highly cluttered airspace	at swarming sites, in hibernacula	predominantly August - September, also March - April	in flight	sedentary	[12, 13]
E. nilssonii	Open- and edge-space forager, aerial hawking in uncluttered or background-cluttered airspace	at swarming sites, in hibernacula	August - September	unknown	sedentary	[14, 15]
E. serotinus	Open- and edge-space forager, aerial hawking in uncluttered or background-cluttered airspace	probably at swarming sites, probably in hibernacula	probably August - October	unknown	sedentary	[16, 17]

* Niche terminology adapted from [18, 19]. † Data from [20]. ‡ General data from [21, 22, 23, 24, 25].

Supplementary Table 6. Flight speed and distance covered during song events recorded at wind turbines.

Species	Song Event					Average flight speed [m/s]	References for flight speed (of commuting bats)	Distance covered during average song event [m]
	Average duration [s]	Median duration [s]	Min. duration [s]	Max. duration [s]	Total number			
N. leisleri	29	29	29	29	1	5.8	[26]	168.2
N. noctula	11.8	6	2	60	38	6	[27]	70.6
P. nathusii	15.0	8	2	92	22	5.1	[28] cited in [29]	76.3
P. pipistrellus	33.2	29	6	62	6	5.5	[30]	182.4
P. pygmaeus	25.4	14	5	71	5	5.5	no reference available *	139.7
Plecotus spp.	2.0	2	2	2	2	2.5	[31]	5.0
V. murinus	27.8	14	2	98	14	5.5	[32]	152.8

* We assumed *P. pygmaeus* had a flight speed comparable to *P. pipistrellus* because no data was available for *P. pygmaeus.*

Supplementary Table 7. Average detection ranges of bat song using the Avisoft detector system (USG 116Hnbm with a Knowles, FG23629-P16 microphone) operated with a trigger level threshold of 37 dB SPL and assuming a temperature of 20° Celsius and 60% relative humidity.

Species	Song peak freq [kHz] *	References for song peak freq	Call intensity [dB peSPL at 1m] †	References for call intensity	Average detection range of song [m]
V. murinus	13.2	[21]	107 ‡	[33]	114.3
N. noctula	26.4	[21]	108	[33]	50.9
N. leisleri	22.3	[21]	104	[33]	57.8
P. nathusii	18.5	[21]	107	[33]	79.9
P. pipistrellus	18.1	[21]	105	[33]	78.3
P. pygmaeus	21.8	[21]	103	[33]	58.0
Plecotus spp.	14.0	[13]	72	[34]	25.1

Supplementary Table 8. Average, minimum and maximum detection ranges of the Avisoft detector system (USG 116Hnbm with a Knowles, FG23629-P16 microphone) operated with a trigger level threshold of 37 dB SPL and assuming a bat call with a sound pressure level of 120 dB at a distance of 10 cm which corresponds to 100 dB at a distance of 1 m and 60% relative humidity.

Frequency (kHZ)	Temperature (°C)	Average detection range (m)	Minimum detection range (m)	Maximum detection range (m)
20	15	64.6	59.4	67.7
40	15	25.6	21.9	28.2
55	15	21.8	18.8	23.7
20	20	**70.9**	65.2	74.4
40	20	23.9	20.4	26.3
55	20	19.0	16.5	20.6
20	25	81.7	74.9	85.9
40	25	24.5	21.0	27.0
55	25	18.1	15.8	19.7

The detection range of a recorder is dependent on several variables such as the sound pressure level and the frequency of the bat call, the frequency response and directivity of the microphone and geometric and atmospheric attenuation of ultrasound in air, the latter of which are variable and determined by whether conditions [33, 35, 36, 37, 38, 39]. We estimated the minimum, maximum and average detection distances as explained in [40] and [39] for weather conditions typically encountered at the studied wind turbines. Most bat activity (approx. 80%) at wind turbines was recorded for temperatures between 15 °C and 25°C [40]. We used the average detection distance at 20°C and 60 % humidity for a vocalization at 20 kHz (i.e. bat song peak frequencies: 14 kHz - 26 kHz) with a trigger threshold level of 37 dB SPL as a proxy for the maximum distance a singing bat was able to cover without leaving the detection range of the acoustic recorder (highlighted in bold in the table).

Supplementary Table 9. Parameter values used to calculate the acoustic signaling range of bat songs and the average detection range of songs by the Avisoft detector system (USG 116Hnbm with a Knowles, FG23629-P16 mircophone).

Species	Song peak freq [kHz] *	References for song peak freq	Call intensity [dB peSPL at 1m] †	References for call intensity	Dynamic range [dB peSPL] §	Max. detection distance of objects by echolocation [m]	Active space of song [m] ¦¦
V. murinus	13.2	[21]	107 ‡	[33]	87	50.1	100.2 (185.2)
N. noctula	26.4	[21]	108	[33]	88	34.7	69.4
N. leisleri	22.3	[21]	104	[33]	84	40.7	81.4
P. nathusii	18.5	[21]	107	[33]	87	50.1	100.2 (112.2)
P. pipistrellus	18.1	[21]	105	[33]	85	48.3	96.6 (111.6)
P. pygmaeus	21.8	[21]	103	[33]	83	41.2	82.4
Plecotus spp.	14.0	[13]	72	[34]	52	20.9	41.8 (63.6)

* Song peak frequencies below 20 kHz were considered to equal 20 kHz since [41] did not model frequencies below 20 kHz. However, in the last column, we report (in brackets) also the active space calculated from the real peak frequencies.

† Unpublished evidence from free-flying bats suggests that song intensities correspond to the species-specific intensity of echolocation calls (pers. communication, Karl-Heinz Frommolt).

‡ Call intensity of *Eptesicus serotinus* was used because no information for *Vespertilio murinus* was available.

§ Dynamic range = call intensity at 1m - detection threshold. Following [41], we assumed a detection threshold of 20 dB.

¦¦ We doubled the calculated max. detection distances because song echoes do not need to travel back to the emitter but are picked up by a receiver instead.

Supplementary Table 10. References for species identification of echolocation calls, social calls, songs and song flight behavior

Species	Additional * references for social calls, songs, and song flight behavior
V. murinus	[1, 20]
N. noctula	[3, 42, 43, 44]
N. leisleri	[5, 6, 45]
P. nathusii	[7, 8, 46]
P. pipistrellus	[9, 10, 47, 48]
P. pygmaeus	[10, 47, 49]
Plecotus spp.	[12, 13, 50, 51]

* Species identification of echolocation call sequences, social calls and songs was based on [21], [23], [25], [52], and [53]. Additional references for social calls, songs, and songflight behavior are listed above.

Supplementary Table 11. Details of the 3D thermal imaging data set

Wind turbine	Date	Recoding time	Total number of recorded flight trajectories	Number of flight trajectories within radius < 60 m	Number of flight trajectories within radius < 30m
D12a	27./28.08.08	21:10 - 03:20	157	41	26
D12a	28./29.08.08	21:05 - 00:00	50	11	5
D1418m	9./10.08.12	21:08 - 03:15	22	5	2
D1418n	10./11.08.12	21:03 - 02:18	52	14	6
D12a	18./19.09.12	19:41 - 00:16	53	9	4
D12b	19./20.09.12	19:22 - 00:12	64	9	1
**Sum flight trajectories**			**398**	**89**	**44**
**Number of bat positions**				**4468**	**1489**

Supplementary Table 12. Comparison between optical recording of flight trajectories and acoustic detection of bats at the sampled wind turbines.

Closest approach to wind turbine	Number of 3D flight tracks	Number of acoustic bat detections *	Probability of acoustic recording (%)
0m - < 10m	5	3	60
10m - < 20m	16	13	81
20m - < 30m	23	6	26
30m - < 40m	26	6	23
40m - < 50m	18	1	6
≥ 50m	164	0	0
**Sum**	**252**	**32**	**13**

* Bat calls were recorded with several detector systems (Anabat, Batcorder, and Avisoft UltraSoundGate) that were installed in the nacelles of wind turbines during thermal imaging. Most recorded flight trajectories approaching the nacelle to less than 20 m were confirmed as bats by acoustic recordings (76%). Acoustic detection probability decreased significantly for flight trajectories approaching the nacelle to 20m – 50m (6-26%). For flight trajectories at distances of ≥ 50m to the nacelle no bats were recorded acoustically. These detection probabilities are well in line with the detection ranges of the used detector systems. At a distance of approximately 20 m to the nacelle, Nyctaloid (16-30 kHz) and Pipistrelloid (37 – 60 kHz) echolocation calls will trigger recordings, whereas at distances larger than 20m the probability of recording bats, especially such with higher frequency echolocation calls decreases considerably with increasing distance to the nacelle. Therefore, and to not bias our data analysis for flight trajectories recorded closer to wind turbines we assumed that all recorded flight trajectories were from flying bats when calculating bat density around the nacelle.

Supplementary References

1. Zagmajster M. Display song of parti-coloured bat *Vespertilio murinus* Linnaeus, 1758 (Chiroptera, Mammalia) in southern Slovenia and preliminary study of its variability. *Natura Sloveniae* **5**, 27–41 (2003).

2. Rydell J, Baagøe HJ. *Vespertilio murinus*. *Mammalian Species*, 1–6 (1994).

3. Weid R. Sozialrufe männlicher Abendsegler (*Nyctalus noctula*). *Bonn Zool Beitr* **45**, 33–38 (1994).

4. Barros P, Braz L, Vale-Gonçalves HM, Cabral JA. First records of *Nyctalus noctula* social calls in Portugal. *Vespertilio* **17**, 37–44 (2014).

5. Ohlendorf B, Ohlendorf L. Zur Wahl der Paarungsquartiere und zur Struktur der Haremsgesellschaften des Kleinabendseglers (*Nyctalus leisleri*) in Sachsen-Anhalt. *Nyctalus (NF)* **6**, 476–491 (1998).

6. Zingg P. Search calls of echolocating *Nyctalus leisleri* and *Pipistrellus savii* (Mammalia: Chiroptera) recorded in Switzerland. *Z Säugetierkd* **53**, 281–293 (1988).

7. Russ JM, Racey PA. Species-specificity and individual variation in the song of male Nathusius' pipistrelles (*Pipistrellus nathusii*). *Behavioral Ecology and Sociobiology* **61**, 669–677 (2007).

8. Jahelkova H, Horacek I, Bartonicka T. The advertisement song of *Pipistrellus nathusii* (Chiroptera, Vespertilionidae): a complex message containing acoustic signatures of individuals. *Acta Chiropt* **10**, 103–126 (2008).

9. Sachteleben J, von Helversen O. Songflight behaviour and mating system of the pipistrelle bat (*Pipistrellus pipistrellus*) in an urban habitat. *Acta Chiropt* **8**, 391–401 (2006).

10. Barlow KE, Jones G. Differences in songflight calls and social calls between two phonic types of the vespertilionid bat *Pipistrellus pipistrellus*. *J Zool* **241**, 315–324 (1997).

11. Bartoničková L, Reiter A, Bartonička T. Mating and Courtship Behaviour of Two Sibling Bat Species *Pipistrellus pipistrellus*, *P. pygmaeus* in the Vicinity of a Hibernaculum. *Acta Chiropt* **18**, 467–475, 469 (2016).

12. Furmankiewicz J, Duma K, Manias K, Borowiec M. Reproductive Status and Vocalisation in Swarming Bats Indicate a Mating Function of Swarming and an Extended Mating Period in *Plecotus auritus*. *Acta Chiropt* **15**, 371–385, 315 (2013).

13. Furmankiewicz J. Social calls and vocal activity of the brown long-eared bat *Plecotus auritus* in SW Poland. *Rhinolophe* **17**, 101–120 (2005).

14. Rydell J. *Eptesicus nilssonii*. *Mammalian Species*, 1–7 (1993).

15. Smirnov DG, Baishev FZ, Bezrukov VA, Vekhnik VP, Kurmaeva NM. The Spatial-Genetic Population Structure of *Eptesicus nilssonii* (Chiroptera, Vespertilionidae) on the Southern Border of Its Range within European Russia. *Biology Bulletin* **47**, 427–439 (2020).

16. Moussy C*, et al.* Population genetic structure of serotine bats (*Eptesicus serotinus*) across Europe and implications for the potential spread of bat rabies (European bat lyssavirus EBLV-1). *Heredity* **115**, 83–92 (2015).

17. Martinoli A, Mazzamuto MV, Spada M. Serotine *Eptesicus serotinus* (Schreber, 1774). In: *Handbook of the Mammals of Europe* (eds Hackländer K, Zachos FE). Springer International Publishing (2020).

18. Schnitzler H-U, Kalko EKV. Echolocation by Insect-Eating Bats: We define four distinct functional groups of bats and find differences in signal structure that correlate with the typical echolocation tasks faced by each group. *Bioscience* **51**, 557–569 (2001).

19. Schnitzler H-U, Moss CF, Denzinger A. From spatial orientation to food acquisition in echolocating bats. *Trends in Ecology & Evolution* **18**, 386–394 (2003).

20. Dietz C, von Helversen O, Nill D. *Handbuch der Fledermäuse Europas und Nordwestafrikas: Biologie, Kennzeichen, Gefährdung*. Kosmos (2007).

21. Pfalzer G. Inter-und intraspezifische Variabilität der Soziallaute heimischer Fledermausarten (Chiroptera: Vespertilionidae).). University of Kaiserslautern, Germany (2002).

22. Pfalzer G, Kusch J. Structure and variability of bat social calls: implications for specificity and individual recognition. *J Zool* **261**, 21–33 (2003).

23. Skiba R. *Europäische Fledermäuse. Kennzeichen, Echoortung und Detektoranwendung*. Neue Brehm Bücherei, Bd 648 (2003).

24. Russ J. *Bat Calls of Britain and Europe: A Guide to Species Identification*. Pelagic Publishing (2021).

25. Middleton N, Froud A, French K. *Social Calls of the Bats of Britain and Ireland: Expanded and Revised Second Edition.* Pelagic Publishing (2022).

26. Shiel CB, Shiel RE, Fairley JS. Seasonal changes in the foraging behaviour of Leisler's bats (*Nyctalus leisleri*) in Ireland as revealed by radio-telemetry. *J Zool* **249**, 347–358 (1999).

27. Jones G. Flight performance, echolocation and foraging behaviour in noctule bats *Nyctalus noctula*. *J Zool* **237**, 303–312 (1995).

28. Baagøe H. The Scandinavian bat fauna: adaptive wing morphology and free flight in the field. In: *Recent Advances in the Study of Bats* (eds Fenton M, Racey P, Rayner J). Cambridge University Press (1987).

29. Šuba J. Migrating Nathusius ' s pipistrelles Pipistrellus nathusii (Chiroptera : Vespertilionidae) optimise flight speed and maintain acoustic contact with the ground. *Environmental and Experimental Biology* **12**, 7–14 (2014).

30. Schaub A, Schnitzler H-U. Flight and echolocation behaviour of three vespertilionid bat species while commuting on flyways. *J Comp Physiol, A* **193**, 1185–1194 (2007).

31. Henningsson P, Jakobsen L, Hedenström A. Aerodynamics of manoeuvring flight in brown long-eared bats *Plecotus auritus*. *J R Soc Interface* **15**, 20180441 (2018).

32. Moeschler P, Blant J-D. *Vespertilio murinus*. In: *Säugetiere der Schweiz / Mammifères de la Suisse / Mammiferi della Svizzera: Verbreitung · Biologie · Ökologie / Répartition · Biologie · Ecologie / Distribuzione · Biologia · Ecologia* (ed Hausser J). Birkhäuser Basel (1995).

33. Holderied MW, von Helversen O. Echolocation range and wingbeat period match in aerial-hawking bats. *Proc R Soc Lond B Biol Sci* **270**, 2293–2299 (2003).

34. Waters DA, Jones G. Echolocation call structure and intensity in five species of insectivorous bats. *J Exp Biol* **198**, 475–489 (1995).

35. Bass HE, Sutherland LC, Zuckerwar AJ. Atmospheric absorption of sound: Update. *Journal of the Acoustical Society of America* **88**, 2019–2021 (1990).

36. Bass HE, Sutherland LC, Zuckerwar AJ, Blackstock DT, Hester DM. Erratum: Atmospheric absorption of sound: Further developments [J. Acoust. Soc. Am. 97, 680–683 (1995)]. *Journal of the Acoustical Society of America* **99**, 1259–1259 (1996).

37. Evans LB, Bass HE, Sutherland LC. Atmospheric Absorption of Sound: Theoretical Predictions. *Journal of the Acoustical Society of America* **51**, 1565–1575 (1972).

38. Goerlitz HR. Weather conditions determine attenuation and speed of sound: Environmental limitations for monitoring and analyzing bat echolocation. *Ecol Evol* **8**, 5090–5100 (2018).

39. Hochradel K. Detection and Localization of Bats.). UMIT - Private University for Health Sciences, Medical Informatics and Technology GmbH (2020).

40. Behr O*, et al.* Akustische Erfassung der Fledermausaktivität. In: *Reduktion des Kollisionsrisikos von Fledermäusen an Onshore-Windenergieanlagen (RENEBAT II)* (eds Behr O*, et al.*). Umwelt und Raum (2015).

41. Stilz W-P, Schnitzler H-U. Estimation of the acoustic range of bat echolocation for extended targets. *Journal of the Acoustical Society of America* **132**, 1765–1775 (2012).

42. Zingg PE. Eine auffällige Lautäusserung des Abendseglers, *Nyctalus noctula* (Schreber) zur Paarungszeit (Mammalia: Chiroptera). *Revue suisse de Zoologie* **1057**, 1062 (1988).

43. Thode JA. Untersuchungen zum Balzgesang des Großen Abendseglers (*Nyctalus noctula*).). FU Berlin (2010).

44. Maas D. Zur akustischen Balzaktivität des Großen Abendseglers (*Nyctalus noctula*): Die Vokalisation der Abendseglermännchen im Verlauf einer Balzsaison.). FU Berlin (2010).

45. von Helversen O, von Helversen D. The advertisement song of the lesser noctule bat (*Nyctalus leisleri*). *Folia Zoologica* **43**, 331–338 (1994).

46. Jahelková H, Horáček I. Mating System of a Migratory Bat, Nathusius' Pipistrelle (*Pipistrellus nathusii*): Different Male Strategies. *Acta Chiropt* **13**, 123–137, 115 (2011).

47. Barlow KE, Jones G. Function of pipistrelle social calls: field data and a playback experiment. *Animal Behaviour* **53**, 991–999 (1997).

48. Budenz T, Heib S, Kusch J. Functions of Bat Social Calls: The Influence of Local Abundance, Interspecific Interactions and Season on the Production of Pipistrelle *Pipistrellus pipistrellus* Type D Social Calls. *Acta Chiropt* **11**, 173–182, 110 (2009).

49. Lundberg K, Gerell R. Territorial Advertisement and Mate Attraction in the Bat *Pipistrellus pipistrellus*. *Ethology* **71**, 115–124 (1986).

50. Furmankiewicz J. The Social Organization and Behavior of the Brown Long-Eared Bat *Plecotus auritus*. In: *Sociality in Bats* (ed Ortega J). Springer International Publishing (2016).

51. Meineke T. Jahres- und tageszeitliche Phänologie rufaktiver Grauer Langohren *Plecotus austriacus* auct. an einem Wohnhaus im südlichen Niedersachsen. *Säugetierkundliche Informationen* **Jena 11**, 11–33 (2018).

52. Barataud M. *Acoustic ecology of European bats. Species Identification and Studies of Their Habitats and Foraging Behaviour. Biotope Editions, Mèze*. National Museum of Natural History (2015).

53. Pfeiffer B, Marckmann U. Bestimmung von Fledermausrufaufnahmen und Kriterien für die Wertung von akustischen Artnachweisen. Teil 1 – Gattungen *Nyctalus*, *Eptesicus*, *Vespertilio*, *Pipistrellus* (nyctaloide und pipistrelloide Arten), Mopsfledermaus, Langohrfledermäuse und Hufeisennasen Bayerns.). Bayerisches Landesamt für Umwelt (LfU) (2020).
