# Supplementary material for: Song flight and 3D thermal detection provide evidence for bat attraction to wind turbines in Central Europe
Source: Commun Biol. 2026 Mar 19;9:460. doi: 10.1038/s42003-026-09882-7 (PMC13032924; doi:10.1038/s42003-026-09882-7)
Supplement: Supplementary file 2 — Reporting Summary [file 42003_2026_9882_MOESM2_ESM.pdf]

nature portfolio

Corresponding author(s): Martina Nagy

Last updated by author(s): Jan 30, 2026

Reporting Summary

Nature Portfolio wishes to improve the reproducibility of the work that we publish. This form provides structure for consistency and transparency in reporting. For further information on Nature Portfolio policies, see our Editorial Policies and the Editorial Policy Checklist.

Statistics

For all statistical analyses, confirm that the following items are present in the figure legend, table legend, main text, or Methods section.

n/a	Confirmed	
☐	☒	The exact sample size (*n*) for each experimental group/condition, given as a discrete number and unit of measurement
☐	☒	A statement on whether measurements were taken from distinct samples or whether the same sample was measured repeatedly
☐	☒	The statistical test(s) used AND whether they are one- or two-sided<br>*Only common tests should be described solely by name; describe more complex techniques in the Methods section.*
☐	☒	A description of all covariates tested
☐	☒	A description of any assumptions or corrections, such as tests of normality and adjustment for multiple comparisons
☐	☒	A full description of the statistical parameters including central tendency (e.g. means) or other basic estimates (e.g. regression coefficient) AND variation (e.g. standard deviation) or associated estimates of uncertainty (e.g. confidence intervals)
☒	☐	For null hypothesis testing, the test statistic (e.g. *F*, *t*, *r*) with confidence intervals, effect sizes, degrees of freedom and *P* value noted<br>*Give P values as exact values whenever suitable.*
☒	☐	For Bayesian analysis, information on the choice of priors and Markov chain Monte Carlo settings
☐	☒	For hierarchical and complex designs, identification of the appropriate level for tests and full reporting of outcomes
☒	☐	Estimates of effect sizes (e.g. Cohen's *d*, Pearson's *r*), indicating how they were calculated

Our web collection on statistics for biologists contains articles on many of the points above.

Software and code

Policy information about availability of computer code

Data collection	We used the RECORDER software (Avisoft Bioacoustics, Glienike, Germany) to continuously record acoustic bat activity at wind turbines. We used Quantum Well Infrared Photodetector cameras (AIM 640Q, AIM Infrarot-Module GmbH) to record 3D flight trajectories of bats at wind turbines.
Data analysis	We used the RECORDER software (Avisoft Bioacoustics, Glienike, Germany) with the 'RENEBAT-settings' to automatically identify bat calls within acoustic recordings and to identify bat species groups. We used the software Avisoft SASLab Pro v5.2.13 and v5.2.14 for sound analysis and to manually identify feeding buzzes and social vocalizations of bats. We calculated the acoustic signaling range of bat songs with a formula developed by Stilz and Schnitzler (http://u-017-s090.v263.uni-tuebingen.de/~peter/open/calculator/range.php). 3D positions of bats were calculated using LabVIEW (National Instruments, Austin, TX) and MATLAB (version 2012b, Mathworks, Inc., Natick, MA, USA). Statistical analysis was conducted in R (https://www.r-project.org/).

For manuscripts utilizing custom algorithms or software that are central to the research but not yet described in published literature, software must be made available to editors and reviewers. We strongly encourage code deposition in a community repository (e.g. GitHub). See the Nature Portfolio guidelines for submitting code & software for further information.

Data

Policy information about availability of data

All manuscripts must include a data availability statement. This statement should provide the following information, where applicable:

- Accession codes, unique identifiers, or web links for publicly available datasets
- A description of any restrictions on data availability
- For clinical datasets or third party data, please ensure that the statement adheres to our policy

The data that support the findings of this study are available in the supporting information and/or will be made available in an open access repository (e.g. data dryad).

Research involving human participants, their data, or biological material

Policy information about studies with human participants or human data. See also policy information about sex, gender (identity/presentation), and sexual orientation and race, ethnicity and racism.

Reporting on sex and gender	n/a
Reporting on race, ethnicity, or other socially relevant groupings	n/a
Population characteristics	n/a
Recruitment	n/a
Ethics oversight	n/a

Note that full information on the approval of the study protocol must also be provided in the manuscript.

Field-specific reporting

Please select the one below that is the best fit for your research. If you are not sure, read the appropriate sections before making your selection.

☐ Life sciences ☐ Behavioural & social sciences ☒ Ecological, evolutionary & environmental sciences

For a reference copy of the document with all sections, see nature.com/documents/nr-reporting-summary-flat.pdf

Ecological, evolutionary & environmental sciences study design

All studies must disclose on these points even when the disclosure is negative.

Study description	1. Acoustic recordings of bats at nacelle height of wind turbines.<br>2. Stereo thermal video surveillance of flight trajectories of bats at wind turbines.
Research sample	1. Acoustic data were recorded at 22 wind turbines (2-4 turbines per site) and at six sites representing four major landscape units of Germany.<br>2. We monitored 4 turbines with stereo-thermal video surveillance cameras during six nights and 30 hours.
Sampling strategy	1. Sample size was determined by the availability of continuously recorded acoustic data from all wind turbines monitored within the RENEBAT II and III projects during the bat activity season. All recordings that met predefined quality criteria were included; no a priori power analysis was performed because this was a retrospective analysis of an existing large-scale monitoring dataset.<br>2. Stereo-thermal recordings were collected during all feasible nights at selected turbines, constrained by weather, logistics and camera availability. The resulting dataset represents the complete set of usable recordings rather than a predefined target sample size.
Data collection	1. Acoustic data was collected by custom hardware and software. Enercon service teams installed detector systems ((Avisoft Bioacoustics, Glienike, Germany) in the nacelle of each turbine to continuously record bat activity in the rotor swept area of wind turbines. Acoustic data collection was coordinated and overseen by MN, OB and RS.<br>2. Stereo thermal video recordings were collected by the Fraunhofer Institute of Optronics, System Technologies and Image Exploitation (IOSB) and by KH.
Timing and spatial scale	1. Detectors were installed at the nacelle of wind turbines between April and beginning of July in 2012 (n=6 wind turbines), 2014 (n=16 wind turbines) and 2015 (n=4 wind turbines) and were run continuously until September to December, covering the main activity period of bats in Central Europe from July to September. Detectors ran continuously but produced valid data only during 94 % of the nights sampled due to detector downtimes (i.e., power or microphone failures or other technical problems). The mean number of nights with valid data for 26 turbine years was 170 ± 40 (Range 76 – 210 nights) of a total of 181 ± 39 nights sampled per

	turbine year (Range 112-228 nights). Acoustic data was recorded at six sites in Germany representing four major landscape units of Germany. Figure S12 shows of map of the natural regions where wind turbines were located. The exact locations of the wind turbines cannot be disclosed as the operators of the wind turbines have been contractually guaranteed the anonymization of the data.<br>2. Four wind turbines were monitored with stereo-thermal video surveillance cameras during six nights and a total of 30 hours in 2008 and 2012. The exact locations of the wind turbines cannot be disclosed as the operators of the wind turbines have been contractually guaranteed the anonymization of the data.
Data exclusions	1. 6% of acoustic recording time could not be used due to detector downtimes (i.e., power or microphone failures or other technical problems). For acoustic GLMM analyses, wind turbine-year-month observations with zero bat recordings were excluded (11 observations), resulting in 267 included observations. Inclusion of these zero-count observations did not affect model estimates.<br>2. No stereo thermal trajectories were excluded.
Reproducibility	Independent datasets from multiple turbines, turbine sites and years were analysed using identical methods.
Randomization	n/a
Blinding	Data analysis was not blinded, but acoustic data was analysed by multiple experienced and unexperienced analysts independently.

Did the study involve field work? ☒ Yes ☐ No

Field work, collection and transport

Field conditions	Acoustic recordings started between April and beginning of July and were run continuously until September to December during all weather conditions. Data collection for stereo thermal video surveillance was performed on rather warm nights with low wind.
Location	Acoustic data were recorded at six sites belonging to four major landscape units of Germany (D02 Northeast Mecklenburg Plain (site 1), D14 Upper Lusatia (site 2), D18 Thuringian Basin (sites 3-5), D52 Saar-Nahe Hills (site 6)) . The exact locations of the wind turbines cannot be disclosed as the operators of the wind turbines have been contractually guaranteed the anonymization of the data.<br>Stereo thermal recordings were performed at two sites (Brandenburg Heath and Lake District (D12) and Thuringian Basin (D18))
Access & import/export	No permission was required for acoustic and thermal recordings from German authorities. Access to the wind turbines for installing recording equipment was granted by the operators of wind turbines.
Disturbance	Data recording was noninvasive and took place below or from within the nacelle of wind turbines. Therefore, no disturbance was made to bats. No handling or manipulation of animals occured.

Reporting for specific materials, systems and methods

We require information from authors about some types of materials, experimental systems and methods used in many studies. Here, indicate whether each material, system or method listed is relevant to your study. If you are not sure if a list item applies to your research, read the appropriate section before selecting a response.

Materials & experimental systems

n/a	Involved in the study	
☒	☐	Antibodies
☒	☐	Eukaryotic cell lines
☒	☐	Palaeontology and archaeology
☐	☒	Animals and other organisms
☒	☐	Clinical data
☒	☐	Dual use research of concern
☒	☐	Plants

Methods

n/a	Involved in the study	
☒	☐	ChIP-seq
☒	☐	Flow cytometry
☒	☐	MRI-based neuroimaging

Animals and other research organisms

Policy information about studies involving animals; ARRIVE guidelines recommended for reporting animal research, and Sex and Gender in Research

Laboratory animals	The study did not involve laboratory animals.
Wild animals	The study involved free-flying wild bats monitored using non-invasive acoustic detectors and stereo-thermal cameras. No animals were captured, handled, marked, or manipulated.
Reporting on sex	Sex of individual bats could generally not be determined because the bats were not captured or handled, and sex cannot be reliably inferred from acoustic or thermal recordings. The only exception concerns acoustic recordings of song, which were attributed to

	male bats based on established evidence that song production in the studied species is restricted to males in territorial or courtship contexts.
Field-collected samples	The study did not involve animals collected from the field.
Ethics oversight	The study involved non-invasive recording of free-flying bats and did not involve capture, handling or experimental manipulation of animals. Therefore, no animal ethics approval or permits were requiered under German regulations.

Note that full information on the approval of the study protocol must also be provided in the manuscript.

Plants

Seed stocks	n/a
Novel plant genotypes	n/a
Authentication	n/a
